# Risk factors for postoperative pulmonary complications in esophageal cancer: focus on inflammatory markers

**DOI:** 10.3389/fonc.2026.1763832

**Published:** 2026-02-24

**Authors:** Mengchao Xue, Ruihao Zhang, Yirou Ma, Yiyang Liu, Junjie Liu, Zhenyi Li, Bingtao Huang, Zheng Ma, Ming Lu, Yongxin Zhou

**Affiliations:** 1 Tongji Hospital, School of Medicine, Tongji University, Shanghai, China; 2 Qilu Hospital of Shandong University, Jinan, China; 3 Binzhou Medical University Affiliated Hospital, Binzhou, China

**Keywords:** esophagectomy, inflammatory factors, LMR, postoperative pulmonary complications, risk factors

## Abstract

**Background:**

In patients with esophageal cancer (EC), postoperative pulmonary complications (PPCs) have an impact on both the long-term prognosis and postoperative recovery. The prognostic utility of novel inflammatory biomarkers for PPCs is yet unknown, despite the fact that systemic inflammation is a hallmark of malignancy. The objective of this study was to methodically identify perioperative parameters that are independently linked to the formation of PPCs, with an emphasis on inflammatory markers.

**Methods:**

781 individuals receiving elective EC resection between January 2022 and December 2024 were included in this retrospective, single-center cohort analysis. Patients were divided into two groups at random: a validation set (n = 232) and a training set (n = 549). To find independent factors linked to PPCs, univariate and multivariate logistic regression analyses were carried out. A nomogram based on the factors found was created for exploratory purposes, and its effectiveness was evaluated.

**Results:**

11.7% of people had PPCs overall. Five independent predictors were found by multivariate analysis: the eosinophil count (OR = 5.924, p=0.027), intraoperative pleural metastasis (OR = 6.853, p=0.026), postoperative ICU admission(OR = 6.963, p=0.006), and postoperative anastomotic leakage (OR = 13.454, p=0.000) were found to be significant risk factors, while the LMR (OR = 0.791, p=0.021) was a protective factor. Limited discriminative ability was demonstrated by the exploratory nomogram based on these parameters (AUC 0.665 in training, 0.561 in validation sets). With modified C-statistics of 0.666 and 0.557 for the training and validation sets, respectively, the DCA showed acceptable discriminatory performance. The DCA revealed a clinically net advantage throughout a broad range of threshold probabilities, and the model demonstrated satisfactory calibration (Hosmer-Lemeshow test p>0.05).

**Conclusion:**

Clinical parameters and inflammatory biomarkers are identified as independent risk factors for PPCs in EC patients. Higher LMR is a protective factor for PPCs, while postoperative ICU admission, higher eosinophil counts, intraoperative pleural metastases, and postoperative anastomotic leaks are risk factors. In order to lower the incidence of PPC, these results offer a theoretical foundation for clinical risk classification and focused preventive measures.

## 1 Introduction

Esophageal cancer (EC) is one of the most aggressive malignant tumors in the world, and its consistently high incidence and death rates present a serious threat to public health across the world (1–4). For patients with resectable EC, a complete treatment plan focusing on radical EC resection remains the most successful therapeutic method (5, 6). However, postoperative problems are common due to the complexity of esophageal surgery (7–11). One of the most prevalent and serious categories of complications among these are postoperative pulmonary complications (PPCs), which include respiratory failure, pneumonia, and acute respiratory distress syndrome. They now have a significant impact on patients’ long-term prognosis and ability to recover (12–18).

Optimizing perioperative care requires identifying patients who are at high risk for PPCs. Several research have tried to develop accurate PPCs predictions (15, 19–23). Traditional predictive models primarily rely on clinical and pathological characteristics, encompassing a wide range of information. This includes preoperative patient data such as age, gender, body mass index (BMI), smoking history, preoperative pulmonary disease, FEV1/FVC ratio, diffusing capacity of the lung for carbon monoxide, white blood cell count, anemia, albumin levels, and history of neoadjuvant therapy. Intraoperative Information: Intraoperative bleeding, type of anesthesia, duration of surgery, surgical technique, pleural adhesions. Postoperative Information: Squamous cell carcinoma, anastomotic leakage and recurrent laryngeal nerve palsy, length of hospital stay, perioperative blood transfusion, T stage, etc. Despite being intimately linked to the incidence of PPCs in clinical practice, key postoperative events (like anastomotic leaks) are rarely consistently included in risk factor studies. This is especially true for the systemic inflammatory response, which is a major factor in postoperative outcomes and a hallmark of malignancy. Finding biomarkers that more accurately capture this pathophysiological milieu and its connection to PPCs is crucial.

Since inflammation is essential to the development and spread of cancer, it is commonly acknowledged as one of its defining characteristics (24–27). In light of this, routine blood test-based systemic inflammatory response indicators have become a class of biomarkers with high clinical usefulness because of their accessibility and affordability (28). For instance, it is widely known that composite indicators like the neutrophil-lymphocyte ratio (NLR), platelet-lymphocyte ratio (PLR), and lymphocyte-monocyte ratio (LMR) are accurate predictors of the host’s systemic inflammatory state (29–31). These ratios provide a strong prognostic ability for EC patients’ long-term survival results (32, 33). However, there is still a substantial research gap: few studies have specifically and methodically investigated the relationship between these easily accessible inflammatory markers and PPCs following EC surgery, nor have they combined preoperative inflammatory indicators with intraoperative and postoperative factors to fully identify independent risk factors for PPCs.

Preoperative inflammatory biomarkers, intraoperative variables, and postoperative key events/indicators are all part of the study’s objective to methodically identify the independent risk factors for postoperative pulmonary problems in EC patients. This study intends to close the current research gap and offer a thorough theoretical foundation for clinical risk stratification and focused preventive interventions by employing rigorous statistical techniques to elucidate the independent associations and possible interactions between these factors and PPCs.

## 2 Methods

### 2.1 Study participants

This research is a retrospective observational study conducted at a single center. Between January 2022 and December 2024, we included 781 patients who had radical resection for EC at Shandong University’s Esophageal Group in the Department of Thoracic Surgery at Qilu Hospital.

The following standards were used to determine which patients were included:

Criteria for Inclusion: (1) Primary EC at postoperative assessment with pathological confirmation; (2) Elective esophageal resection with the goal of curing the disease.

Exclusion criteria include: (1) preoperative distant metastases; (2) emergency surgery; (3) a severe or active pulmonary infection before surgery; (4) a significant clinical documentation gap; and (5) numerous primary malignancies that are concurrent or metachronous.

**Figure 1:**
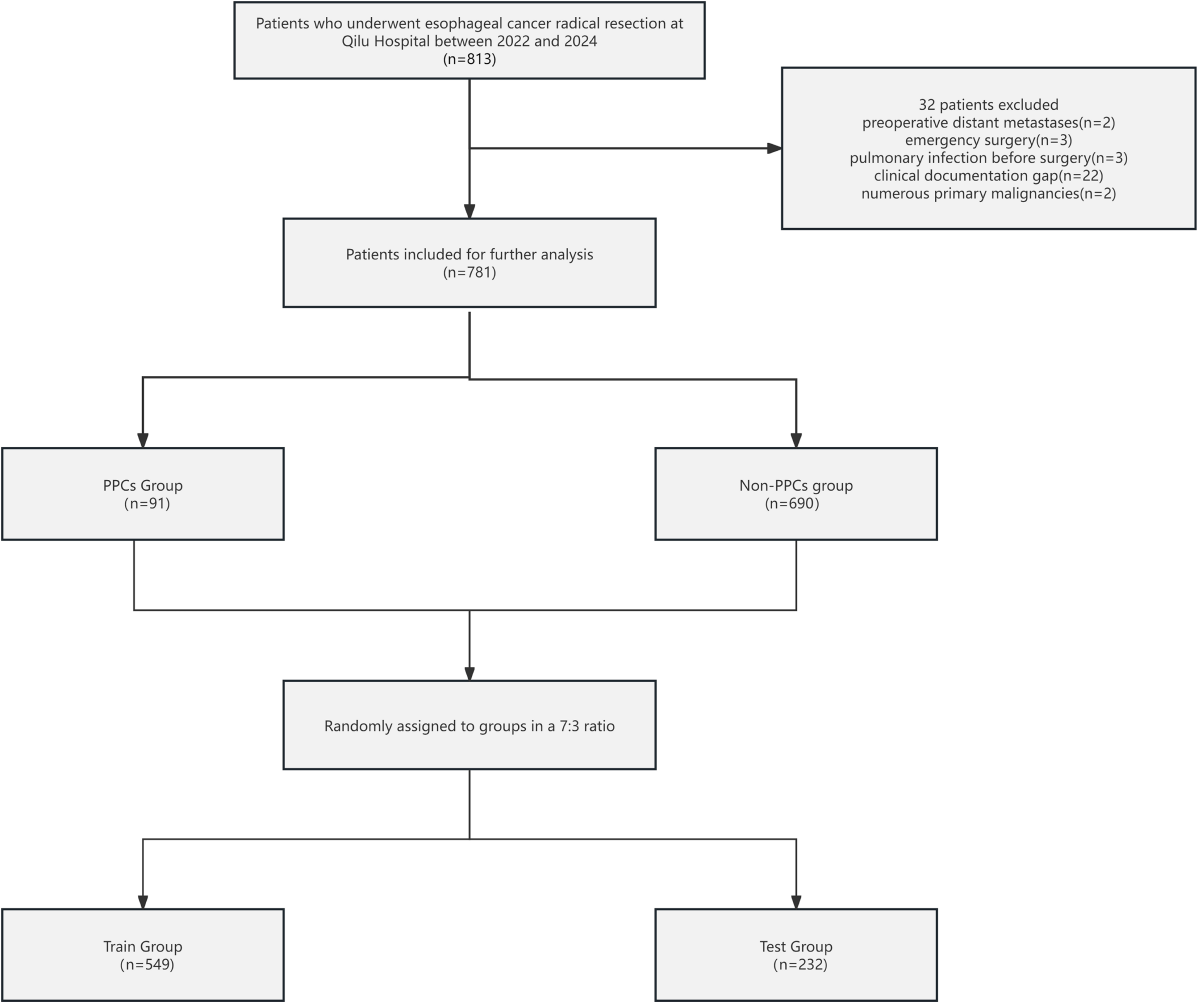
Patient selection flowchart for this study. PPCs, postoperative pulmonary complications.

The Ethics Review Committee of Qilu Hospital, which is affiliated with Shandong University, gave its approval to this research protocol. The Declaration of Helsinki’s ethical guidelines are closely followed in this investigation. The Qilu Ethics Committees approved the investigation (registration number: KYLL-202008-023-1). The Ethics Committee approved an exemption from seeking patients’ individual informed permission due to the study’s retrospective nature. **Figure 1** depicts the comprehensive patient selection procedure.

### 2.2 Perioperative management protocol

Upon admission, all patients go through a thorough preoperative evaluation that includes imaging studies, cardiopulmonary function testing, and a thorough physical examination to rule out distant metastases and finally validate surgical indications. The hospital’s multidisciplinary team must jointly assess all cases that match surgical criteria in order to identify the best course of therapy. Individualized treatment plans will be created by oncology specialists for patients who need neoadjuvant therapy. These could consist of combination immunotherapy, chemoradiation, or chemotherapy.

Double-lumen endotracheal intubation was used for the procedure, which was conducted under general anesthesia. During preoperative planning, the surgical team decides the precise surgical approach depending on the patient’s unique situation and the location of the tumor. Surgical techniques include open and minimally invasive procedures, and access points include incisions in the left or right thorax. Standard two-field dissection, extended two-field dissection, and even three-field dissection are all possible for lymph nodes. The stomach is the preferred esophageal replacement organ during surgery, with the colon or jejunum coming in second or third. The esophageal bed or retrosternal approaches might be used to raise the organ. The neck or the thoracic cavity contain the anastomosis site. Either manual suturing or mechanical stapling are used to perform anastomosis. During surgery, a jejunostomy is performed if the placement of a nasojejunal feeding tube is not effective.

All patients were moved to the surgical critical care unit for one to three days of rigorous monitoring after surgery. On the first postoperative day, enteral nutrition and parenteral support were started, along with physical interventions such back and rotating percussion to encourage the removal of sputum. Patients were returned to the general ward once vital signs had stabilized. Oral feeding is usually started on the seventh postoperative day if anastomotic leaking from the upper gastrointestinal tract has been ruled out. Patients usually heal and are released 10–14 days after surgery if there are no problems such chylothorax, anastomotic leaking, or incisional infection. Patients diagnosed with intraoperative pleural metastasis undergo extended pleurectomy according to institutional guidelines, followed by adjuvant therapy (chemotherapy or immunotherapy), and are closely monitored during follow-up.

### 2.3 Data collection

Through a retrospective analysis of the electronic medical record system, the following four categories of perioperative variables were methodically gathered for this study:

#### 2.3.1 General clinical characteristics

Demographic data, baseline conditions, and comorbidities were collected for patients, including: age, gender, body mass index (BMI), smoking index, and alcohol consumption history; Comorbidities included hypertension, coronary heart disease, diabetes, and family history of cancer; Preoperative treatment history, including surgical history and prior chemotherapy; Functional status assessed using the Karnofsky Performance Status (KPS) score; Maximum voluntary ventilation (MVV), projected MVV, and measured forced expiratory volume in one second (FEV1) are used to assess respiratory function; Records of the patients’ principal clinical symptoms and whether they received preoperative nutritional intervention were also maintained.

#### 2.3.2 Preoperative hematologic and biochemical parameters

A week before surgery, all blood samples were taken. The parameters collected included:

Complete Blood Count Parameters: Neutrophil count, lymphocyte count, eosinophil count, basophil count, monocyte count.

Derived Inflammatory Markers: Calculated based on the above cell counts, including: Neutrophil-to-lymphocyte ratio (NLR), derived NLR (dNLR), lymphocyte-to-monocyte ratio (LMR), platelet-to-lymphocyte ratio (PLR), neutrophil-to-lymphocyte-to-platelet ratio (NLPR), systemic inflammatory response index (SIRI), aggregate immune inflammatory index (AISI), systemic immune inflammatory index (SII), and pan-immune inflammatory value (PIV).

Nutritional and Biochemical Markers: Prognostic Nutritional Index (PNI), Albumin, Total Protein, Albumin-Globulin Ratio, Complement C1q, Lactate Dehydrogenase (LDH), 5’-nucleotidase (5’-NT), total bilirubin, alanine aminotransferase (ALT), aspartate aminotransferase (AST), urea, creatinine, and uric acid.

#### 2.3.3 Surgical variables

Comprehensive documentation of surgical procedures and intraoperative findings, such as anastomosis technique (mechanical/manual), anastomosis site (intrathoracic/cervical), esophageal substitute elevation route (retrosternal/esophageal bed), enteral nutrition access method (nasojejunal tube/jejunostomy), and degree of lymph node dissection (standard two-field/extended two-field/three-field). The existence of pleural adhesions, pleural effusion, ascites, pleural metastases, and whether prolonged resection was necessary because of tumor invasion were among the intraoperative observations that were recorded. The location of the tumor and postoperative care, such as the use of an analgesic pump and ICU stay, were noted. Another important component that was noted was anastomotic leaking.

#### 2.3.4 Postoperative pathological variables

Using the 8th edition of the American Joint Committee on Cancer (AJCC) TNM staging system, all pathology findings were reexamined and verified. Tumor size, degree of tumor resection (R0/R1/R2), T stage, N stage, M stage, G stage (grade), P stage (pathological TNM stage), circumferential margin status, histological type, lymph node metastases status, and presence of vascular/lymphatic invasion were among the variables gathered (34).

We now state that for variables with low missingness (<5%), we used multiple imputation via random forest (using the mice package in R). Variables with missingness >10% were excluded from the analysis.

### 2.4 Variable definition

Pleural metastasis is defined as: macroscopically visible nodules or diffuse implantations on the parietal/visceral pleura observed by the primary surgeon during open thoracotomy or thoracoscopic surgery, confirmed by intraoperative frozen section pathology or definitive postoperative paraffin section pathology. Postoperative pulmonary complications include pneumonia, respiratory failure, acute respiratory distress syndrome (ARDS), clinically significant atelectasis, and massive pleural effusion. The clinical diagnosis of pneumonia is established when chest imaging shows new or increasing pulmonary infiltrates and at least two of the three criteria listed below are present: (1) body temperature >38 °C; (2) purulent airway secretions; (3) peripheral blood white blood cell count >10.0×10^9^/L or <4.0×10^9^/L (35). Respiratory failure is defined as a PaO_2_/FiO_2_ ratio ≤200 mmHg or the need for mechanical ventilation (36). Acute respiratory distress syndrome (ARDS) is diagnosed according to the Berlin criteria (37). Atelectasis is defined as the presence of unilateral or bilateral focal consolidation, indicating focal loss of ventilation. Atelectasis is not included in the case criteria for ARDS, pneumonia, or fluid overload (38). A massive pleural effusion is characterized by respiratory impairment that necessitates closed chest drainage or therapeutic thoracentesis (39).

The number of cigarettes smoked daily times the number of years smoked (cigarette-years) is the smoking index. When patients at nutritional risk get specialist nutritional care (such as enteral or parenteral nutrition or oral nutritional supplements) for at least seven days before surgery, this is referred to as preoperative nutritional intervention.

The following formula is used to determine the derived inflammatory markers:


$$\text{dNLR}\;=\;\text{Absolute Neutrophil Count}\;\left(\times 10^{9}/\text{L}\right)/\left[\text{White Blood Cell Count }\left(\times 10^{9}/\text{L}\right)\;-\;\text{Absolute Neutrophil Count}\left(\times 10^{9}/\text{L}\right)\right].$$

$$\text{NLPR}\;=\;\left[\text{Neutrophil Count }\left(\times 10^{9}/\text{L}\right)\;\times \;\text{Platelet Count}\left(\times 10^{9}/\text{L}\right)\right]\;/\text{ Lymphocyte Count}\left(\times 10^{9}/\text{L}\right).$$

$$\text{SII}\;=\;\left[\text{Platelet Count}\left(\times 10^{9}/\text{L}\right)\;\times \;\text{Neutrophil Count}\;\left(\times 10^{9}/\text{L}\right)\right]/\;\text{Lymphocyte Count}\;\left(\times 10^{9}/\text{L}\right).$$

$$\text{SIRI}\;=\;\left[\text{Neutrophil Count }\left(\times 10^{9}/\text{L}\right)\;\times \;\text{Monocyte Count }\left(\times 10^{9}/\text{L}\right)\right]\;/\;\text{Lymphocyte Count}\;\left(\times 10^{9}/\text{L}\right).$$

$$\text{AISI}\;=\;\left[\text{Neutrophil Count}\;\left(\times 10^{9}/\text{L}\right)\;\times \;\text{Platelet Count}\;\left(\times 10^{9}/\text{L}\right)\;\times \;\;Monocyte\;Count\;\left(\times 10^{9}/\text{L}\right)\right]\;/\;\text{Lymphocyte Count}\;\left(\times 10^{9}/\text{L}\right).$$

$$\text{PIV}\;=\;\left[\text{Neutrophil count }\left(\times 10^{9}/\text{L}\right)\;\times \;\text{Platelet count}\;\left(\times 10^{9}/\text{L}\right)\;\times \;\text{ Monocyte count}\;\left(\times 10^{9}/\text{L}\right)\right]\;/\;\text{Lymphocyte count }\left(\times 10^{9}/\text{L}\right).$$

$$\text{PNI}\;=\;\text{Serum albumin value }\left(\text{g}/\text{L}\right)\;+\;5\;\times \;\text{Absolute lymphocyte count }\left(\times 10^{9}/\text{L}\right).$$

### 2.5 Statistical analysis

This study’s statistical analyses were all conducted using R software (version 4.3.1). Depending on their normality, continuous variables were reported as mean ± standard deviation or median (interquartile range), and the Student’s t-test or Mann-Whitney U test were used for comparison. Categorical variables were presented as frequency (percentage) and compared using chi-square test or Fisher’s exact test.

Patients were divided into training and validation sets at random in a 7:3 ratio. The purpose of this split was to make sure that the risk factors that were found were robust; variables were found in the training set, and their stability was evaluated in the independent validation set. The primary objective was to identify factors independently associated with PPCs. Variables with p-values < 0.05 in univariate analysis were included in the multivariate logistic regression model. Forward stepwise selection was subsequently employed to obtain the final simplified model, thereby identifying independent factors. Results are presented as odds ratios (OR) with their 95% confidence intervals (CI).

Based on the final set of independent factors found in the primary study, a nomogram was created for exploratory reasons. The area under the receiver operating characteristic curve (AUC) and the optimism-corrected C-index (obtained via 1000 bootstrap resamples) were used to evaluate its discriminative capacity. The Hosmer-Lemeshow goodness-of-fit test and calibration plots were used to assess calibration. Decision curve analysis (DCA) was used to investigate clinical utility. SHapley Additive exPlanations (SHAP) analysis was used to display each factor’s contribution and direction of influence in the exploratory model.

## 3 Results

### 3.1 Patient characteristics

In this study, 781 individuals who had radical EC resection were included. 91 patients (11.7%) experienced PPCs (PPCs group), whereas 690 individuals (88.3%) did not (non-PPCs group). **Table 1** provides a detailed comparison of the baseline characteristics of the two groups. Overall, PPCs were associated with significantly lower nutritional immunological indicators (LMR, PNI, albumin-globulin ratio), and significantly higher systemic inflammatory markers (neutrophil count, NLR, dNLR, SIRI). In comparison to the group without PPCs, this group also showed noticeably greater rates of anastomotic fistula formation, postoperative ICU admission, and intraoperative pleural metastases. Age, gender, tumor stage, and histological type were among the baseline parameters that did not show statistically significant variations between the two groups.

**Table 1:** Comparison of the baseline characteristics of the two groups.

Variables	Total (N = 781)	Non-PPCs (N = 690)	PPCs (N = 91)	*p*
Age,Median(Q1,Q3)	63.00(57.50,69.00)	63.00(57.75, 69.00)	65.00(57.00, 71.00)	0.182
Smoking Index,Median(Q1,Q3)	200.00(0.00,600.00)	200.00(0.00, 600.00)	200.00(0.00, 800.00)	0.575
BMI,Median(Q1,Q3)	23.74(21.69,25.82)	23.64(21.64, 25.74)	24.21(22.27, 26.03)	0.235
Actual FEV1 Value,Median(Q1,Q3)	2.78(2.43,3.08)	2.78(2.45, 3.08)	2.76(2.39, 3.06)	0.660
MVV,Median(Q1,Q3)	99.36(80.81,121.48)	99.99(81.38, 121.83)	93.79(78.96, 113.99)	0.190
MVV Predicted Value,Median(Q1,Q3)	99.67(83.88,112.62)	99.75(83.90, 112.60)	97.05(82.61, 113.65)	0.884
Neutrophils,Median(Q1,Q3)	3.65(2.92,4.69)	3.62(2.89, 4.64)	3.93(3.27, 5.12)	0.023
Lymphocyte,Median(Q1,Q3)	1.65(1.29,2.05)	1.66(1.29, 2.06)	1.58(1.29, 1.89)	0.144
Eosinophils,Median(Q1,Q3)	0.10(0.05,0.17)	0.10(0.05, 0.17)	0.10(0.06, 0.16)	0.882
Basophil,Median(Q1,Q3)	0.03(0.02,0.04)	0.03(0.02, 0.04)	0.03(0.02, 0.05)	0.160
Monocyte,Median(Q1,Q3)	0.48(0.39,0.60)	0.47(0.38, 0.60)	0.52(0.40, 0.62)	0.117
PNI,Median(Q1,Q3)	51.35(48.05,54.73)	51.50(48.23, 54.90)	50.10(47.40, 53.25)	0.045
NLR,Median(Q1,Q3)	2.15(1.61,3.02)	2.12(1.60, 2.98)	2.47(1.66, 3.40)	0.030
LMR,Median(Q1,Q3)	3.52(2.73,4.56)	3.61(2.74, 4.60)	3.16(2.55, 4.23)	0.021
PLR,Median(Q1,Q3)	144.28(114.24,185.32)	143.50(113.38, 183.57)	156.03(117.73, 203.12)	0.185
dNLR,Median(Q1,Q3)	1.58(1.23,2.06)	1.55(1.23, 2.03)	1.76(1.26, 2.21)	0.049
NLPR,Median(Q1,Q3)	0.01(0.01,0.01)	0.01(0.01, 0.01)	0.01(0.01, 0.01)	0.053
SIRI,Median(Q1,Q3)	1.01(0.70,1.56)	1.01(0.67, 1.54)	1.19(0.80, 1.93)	0.019
AISI,Median(Q1,Q3)	243.12(149.39,407.73)	239.54(146.47, 400.26)	282.56(181.54, 534.88)	0.054
SII,Median(Q1,Q3)	521.46(360.85,774.96)	517.59(358.30, 754.25)	559.87(394.65, 885.54)	0.090
PIV,Median(Q1,Q3)	243.12(149.39,407.73)	239.54(146.47, 400.26)	282.56(181.54, 534.88)	0.054
Albumin-Globulin Ratio,Median(Q1,Q3)	1.73(1.54,1.92)	1.74(1.54, 1.93)	1.67(1.48, 1.87)	0.046
Complemen C1q,Median(Q1,Q3)	174.90(152.60,197.25)	174.95(152.55, 197.30)	172.90(152.90, 195.60)	0.764
LDH,Median(Q1,Q3)	188.00(167.00,214.00)	188.00(167.00, 214.00)	184.00(170.00, 218.00)	0.780
5’-NT,Median(Q1,Q3)	3.00(3.00,4.00)	3.00(3.00, 4.00)	3.00(3.00, 4.00)	0.804
Total Protein,Median(Q1,Q3)	68.10(64.75,71.20)	68.10(64.80, 71.32)	67.90(64.60, 70.70)	0.398
Albumin,Median(Q1,Q3)	43.10(40.50,45.35)	43.20(40.58, 45.40)	42.40(40.30, 44.20)	0.066
Total Bilirubin,Median(Q1,Q3)	10.00(7.70,13.70)	10.00(7.70, 13.72)	10.30(7.80, 12.60)	0.963
ALT,Median(Q1,Q3)	13.00(10.00,18.00)	13.00(10.00, 18.00)	13.00(10.00, 18.00)	0.431
AST,Median(Q1,Q3)	18.00(15.00,21.00)	18.00(15.00, 21.00)	18.00(15.00, 22.00)	0.924
Urea,Median(Q1,Q3)	4.96(4.16,5.90)	4.96(4.14, 5.87)	5.00(4.17, 6.10)	0.670
Creatinine,Median(Q1,Q3)	71.00(63.00,80.00)	71.00(63.00, 80.00)	73.00(63.00, 81.00)	0.459
Uric Acid,Median(Q1,Q3)	286.00(243.00,333.00)	285.00(242.75, 332.25)	290.00(243.00, 340.00)	0.416
Surgery Duration,Median(Q1,Q3)	180.00(150.00,210.00)	180.00(150.00, 210.00)	180.00(150.00, 235.00)	0.203
Tumor Volume,Median(Q1,Q3)	45.00(24.00,87.00)	46.50(24.00, 84.00)	45.00(24.00, 96.00)	0.920
Gender, n(%)				0.116
Female	131 (16.8%)	121 (17.5%)	10 (11.0%)	
Male	650 (83.2%)	569 (82.5%)	81 (89.0%)	
Preoperative Nutritional Intervention, n(%)				0.468
No	760 (97.3%)	673 (97.5%)	87 (95.6%)	
Yes	21 (2.7%)	17 (2.5%)	4 (4.4%)	
Symptoms, n(%)				0.836
No	11 (1.4%)	9 (1.3%)	2 (2.2%)	
Yes	770 (98.6%)	681 (98.7%)	89 (97.8%)	
Hypertension, n(%)				0.906
No	579 (74.1%)	512 (74.2%)	67 (73.6%)	
Yes	202 (25.9%)	178 (25.8%)	24 (26.4%)	
Diabetes, n(%)				0.251
No	693 (88.7%)	609 (88.3%)	84 (92.3%)	
Yes	88 (11.3%)	81 (11.7%)	7 (7.7%)	
Coronary Heart Disease, n(%)				0.130
No	737 (94.4%)	648 (93.9%)	89 (97.8%)	
Yes	44 (5.6%)	42 (6.1%)	2 (2.2%)	
Surgical History, n(%)				0.130
No	594 (76.1%)	519 (75.2%)	75 (82.4%)	
Yes	187 (23.9%)	171 (24.8%)	16 (17.6%)	
Alcohol History, n(%)				0.261
No	273 (35.0%)	246 (35.7%)	27 (29.7%)	
Yes	508 (65.0%)	444 (64.3%)	64 (70.3%)	
Family History Of Cancer, n(%)				0.749
No	719 (92.1%)	636 (92.2%)	83 (91.2%)	
Yes	62 (7.9%)	54 (7.8%)	8 (8.8%)	
KPS Score, n(%)				0.569
≥ 80	635 (81.3%)	563 (81.6%)	72 (79.1%)	
< 80	146 (18.7%)	127 (18.4%)	19 (20.9%)	
Preoperative Chemotderapy, n(%)				0.905
No	761 (97.4%)	673 (97.5%)	88 (96.7%)	
Yes	20 (2.6%)	17 (2.5%)	3 (3.3%)	
Surgical Procedure, n(%)				0.436
Endoscopic Surgery	260 (33.3%)	233 (33.8%)	27 (29.7%)	
Open Surgery	521 (66.7%)	457 (66.2%)	64 (70.3%)	
Surgical Approach, n(%)				0.071
Left Thoracic Approach	576 (73.8%)	516 (74.8%)	60 (65.9%)	
Right Thoracic Approach	205 (26.2%)	174 (25.2%)	31 (34.1%)	
Anastomosis Technique, n(%)				0.311
Mechanical Anastomosis	778 (99.6%)	688 (99.7%)	90 (98.9%)	
Manual Anastomosis	3 (0.4%)	2 (0.3%)	1 (1.1%)	
Anastomosis Site, n(%)				0.241
Intratdoracic	628 (80.4%)	559 (81.0%)	69 (75.8%)	
Neck	153 (19.6%)	131 (19.0%)	22 (24.2%)	
Elevation Approach, n(%)				0.749
Esophageal Bed	719 (92.1%)	636 (92.2%)	83 (91.2%)	
Retrosternal	62 (7.9%)	54 (7.8%)	8 (8.8%)	
Enteral Nutrition Route, n(%)				0.135
Non	52 (6.7%)	47 (6.8%)	5 (5.5%)	
Nasojejunal tube	680 (87.1%)	604 (87.5%)	76 (83.5%)	
Jejunostomy	49 (6.3%)	39 (5.7%)	10 (11.0%)	
Extent Of Lymph Node Dissection, n(%)				0.055
Standard Two Field	500 (64.0%)	450 (65.2%)	50 (54.9%)	
Above	281 (36.0%)	240 (34.8%)	41 (45.1%)	
Thoracic Adhesions, n(%)				0.309
No	501 (64.1%)	447 (64.8%)	54 (59.3%)	
Yes	280 (35.9%)	243 (35.2%)	37 (40.7%)	
Pleural Effusion, n(%)				0.175
No	687 (88.0%)	603 (87.4%)	84 (92.3%)	
Yes	94 (12.0%)	87 (12.6%)	7 (7.7%)	
Ascites, n(%)				0.409
No	682 (87.3%)	605 (87.7%)	77 (84.6%)	
Yes	99 (12.7%)	85 (12.3%)	14 (15.4%)	
Pleural Metastasis, n(%)				0.008
No	773 (99.0%)	686 (99.4%)	87 (95.6%)	
Yes	8 (1.0%)	4 (0.6%)	4 (4.4%)	
Wide Excision, n(%)				0.691
No	764 (97.8%)	676 (98.0%)	88 (96.7%)	
Yes	17 (2.2%)	14 (2.0%)	3 (3.3%)	
Pain Relief Pump, n(%)				0.026
No	64 (8.2%)	62 (9.0%)	2 (2.2%)	
Yes	717 (91.8%)	628 (91.0%)	89 (97.8%)	
Transfer To ICU, n(%)				<0.001
No	766 (98.1%)	684 (99.1%)	82 (90.1%)	
Yes	15 (1.9%)	6 (0.9%)	9 (9.9%)	
Anastomotic Leakage, n(%)				<0.001
No	764 (97.8%)	682 (98.8%)	82 (90.1%)	
Yes	17 (2.2%)	8 (1.2%)	9 (9.9%)	
Degree of Tumor Resection, n(%)				0.581
R0	774 (99.1%)	684 (99.1%)	90 (98.9%)	
R1+R2	7 (0.9%)	6 (0.9%)	1 (1.1%)	
Tumor Location, n(%)				0.241
Upper	45 (5.8%)	39 (5.7%)	6 (6.6%)	
Middle	311 (39.8%)	268 (38.8%)	43 (47.3%)	
Lower	425 (54.4%)	383 (55.5%)	42 (46.2%)	
T Stage, n(%)				0.677
T1-T2	336 (43.0%)	295 (42.8%)	41 (45.1%)	
T3-T4	445 (57.0%)	395 (57.2%)	50 (54.9%)	
N Stage, n(%)				0.717
N0	383 (49.0%)	340 (49.3%)	43 (47.3%)	
N+	398 (51.0%)	350 (50.7%)	48 (52.7%)	
M Stage, n(%)				1.000
M0	776 (99.4%)	685 (99.3%)	91 (100.0%)	
M1	5 (0.6%)	5 (0.7%)	0 (0.0%)	
G Stage, n(%)				0.170
G1-G2	428 (54.8%)	372 (53.9%)	56 (61.5%)	
G3-G4	353 (45.2%)	318 (46.1%)	35 (38.5%)	
P Stage, n(%)				0.759
I-II	398 (51.0%)	353 (51.2%)	45 (49.5%)	
III-IV	383 (49.0%)	337 (48.8%)	46 (50.5%)	
Circumferential Cut Edge, n(%)				0.526
>1mm	775 (99.2%)	685 (99.3%)	90 (98.9%)	
≤1mm	6 (0.8%)	5 (0.7%)	1 (1.1%)	
Patdological Tissue Type, n(%)				0.492
Squamous cell carcinoma	518 (66.3%)	453 (65.7%)	65 (71.4%)	
Adenocarcinoma	212 (27.1%)	192 (27.8%)	20 (22.0%)	
Otder	51 (6.5%)	45 (6.5%)	6 (6.6%)	
Lymphovascular Invasion, n(%)				0.717
No	580 (74.3%)	511 (74.1%)	69 (75.8%)	
Yes	201 (25.7%)	179 (25.9%)	22 (24.2%)	
Lymph Node Metastasis, n(%)				0.604
No	389 (49.8%)	346 (50.1%)	43 (47.3%)	
Yes	392 (50.2%)	344 (49.9%)	48 (52.7%)	

BMI, body mass index; FEV1, forced expiratory volume in the first second; MVV, maximum voluntary ventilation; PNI, prognostic nutritional index; NLR, neutrophil-to-lymphocyte ratio; LMR, lymphocyte-to-monocyte ratio; PLR, platelet-to-lymphocyte ratio; dNLR, derived neutrophil-to-lymphocyte ratio; NLPR, neutrophil-to-lymphocyte*platelet ratio; SIRI, systemic inflammation response index; AISI, aggregate index of systemic inflammation; SII, systemic immune-inflammation index; PIV, pan-immune-inflammation value; LDH, lactate dehydrogenase; 5’-NT, 5’-nucleotidase; ALT, alanine aminotransferase; AST, aspartate aminotransferase; KPS, karnofsky performance status; ICU, intensive care unit.

This study used random sampling to split all 781 patients into a training set (n=549) and a validation set (n=232), with a sample size ratio of roughly 7:3 between the two groups, in order to ensure the robustness of the identified risk factors. The training set and validation set comparison is displayed in **Table 2**. The two sets were found to have excellent balance across all key variables, including demographic data, clinical features, tumor pathological parameters, surgery-related variables, laboratory indicators, and postoperative complication rates (all P > 0.05). This balanced distribution validates the suitability of the random split and offers a solid foundation for the factor identification and stability evaluation to follow.

**Table 2:** The comparison between the training set and validation set.

Variables	Total (N = 781)	Test data (N = 232)	Trian data (N = 549)	*p*
Age,Median(Q1,Q3)	63.00(57.50,69.00)	63.00(58.00, 68.00)	64.00(57.00, 69.50)	0.300
Smoking Index,Median(Q1,Q3)	200.00(0.00,600.00)	200.00(0.00, 600.00)	200.00(0.00, 600.00)	0.813
BMI,Median(Q1,Q3)	23.74(21.69,25.82)	23.70(21.79, 25.70)	23.78(21.67, 25.93)	0.640
Actual FEV1 Value,Median(Q1,Q3)	2.78(2.43,3.08)	2.80(2.43, 3.07)	2.77(2.43, 3.08)	0.738
MVV,Median(Q1,Q3)	99.36(80.81,121.48)	99.78(82.61, 123.07)	99.33(79.88, 120.24)	0.375
MVV Predicted Value,Median(Q1,Q3)	99.67(83.88,112.62)	102.41(84.00, 113.23)	98.72(83.84, 112.18)	0.437
Neutrophils,Median(Q1,Q3)	3.65(2.92,4.69)	3.70(2.91, 4.68)	3.65(2.92, 4.69)	0.860
Lymphocyte,Median(Q1,Q3)	1.65(1.29,2.05)	1.64(1.30, 2.02)	1.65(1.29, 2.06)	0.973
Eosinophils,Median(Q1,Q3)	0.10(0.05,0.17)	0.09(0.06, 0.17)	0.10(0.05, 0.16)	0.832
Basophil,Median(Q1,Q3)	0.03(0.02,0.04)	0.03(0.02, 0.05)	0.03(0.02, 0.04)	0.188
Monocyte,Median(Q1,Q3)	0.48(0.39,0.60)	0.49(0.39, 0.60)	0.47(0.38, 0.60)	0.245
PNI,Median(Q1,Q3)	51.35(48.05,54.73)	51.35(48.07, 54.50)	51.30(48.02, 54.90)	0.943
NLR,Median(Q1,Q3)	2.15(1.61,3.02)	2.19(1.68, 2.91)	2.13(1.60, 3.06)	0.708
LMR,Median(Q1,Q3)	3.52(2.73,4.56)	3.47(2.61, 4.37)	3.56(2.77, 4.61)	0.244
PLR,Median(Q1,Q3)	144.28(114.24,185.32)	146.32(119.00, 180.66)	143.50(111.14, 188.78)	0.616
dNLR,Median(Q1,Q3)	1.58(1.23,2.06)	1.59(1.23, 2.02)	1.57(1.23, 2.10)	0.828
NLPR,Median(Q1,Q3)	0.01(0.01,0.01)	0.01(0.01, 0.01)	0.01(0.01, 0.01)	0.989
SIRI,Median(Q1,Q3)	1.01(0.70,1.56)	1.03(0.75, 1.63)	1.00(0.67, 1.55)	0.375
AISI,Median(Q1,Q3)	243.12(149.39,407.73)	254.49(156.92, 411.88)	238.56(145.34, 406.91)	0.396
SII,Median(Q1,Q3)	521.46(360.85,774.96)	532.30(372.67, 754.71)	514.94(354.71, 787.09)	0.652
PIV,Median(Q1,Q3)	243.12(149.39,407.73)	254.49(156.92, 411.88)	238.56(145.34, 406.91)	0.396
Albumin-Globulin Ratio,Median(Q1,Q3)	1.73(1.54,1.92)	1.72(1.48, 1.94)	1.74(1.56, 1.92)	0.247
Complemen C1q,Median(Q1,Q3)	174.90(152.60,197.25)	172.65(151.62, 199.65)	175.10(152.75, 196.60)	0.908
LDH,Median(Q1,Q3)	188.00(167.00,214.00)	183.00(165.25, 210.00)	190.00(168.00, 215.00)	0.064
5’-NT,Median(Q1,Q3)	3.00(3.00,4.00)	4.00(3.00, 4.00)	3.00(3.00, 4.00)	0.474
Total Protein,Median(Q1,Q3)	68.10(64.75,71.20)	68.60(65.00, 71.50)	67.90(64.60, 71.05)	0.213
Albumin,Median(Q1,Q3)	43.10(40.50,45.35)	43.10(40.23, 45.68)	43.10(40.60, 45.30)	0.919
Total Bilirubin,Median(Q1,Q3)	10.00(7.70,13.70)	9.70(7.70, 13.57)	10.20(7.80, 13.70)	0.539
ALT,Median(Q1,Q3)	13.00(10.00,18.00)	13.00(9.00, 16.00)	13.00(10.00, 18.00)	0.105
AST,Median(Q1,Q3)	18.00(15.00,21.00)	17.00(15.00, 21.00)	18.00(15.00, 22.00)	0.171
Urea,Median(Q1,Q3)	4.96(4.16,5.90)	4.90(4.12, 5.94)	4.99(4.16, 5.87)	0.872
Creatinine,Median(Q1,Q3)	71.00(63.00,80.00)	72.00(63.00, 79.75)	71.00(63.00, 81.00)	0.884
Uric Acid,Median(Q1,Q3)	286.00(243.00,333.00)	290.50(247.25, 332.00)	285.00(241.00, 333.50)	0.950
Surgery Duration,Median(Q1,Q3)	180.00(150.00,210.00)	180.00(150.00, 210.00)	180.00(150.00, 210.00)	0.445
Tumor Volume,Median(Q1,Q3)	45.00(24.00,87.00)	54.00(24.00, 96.00)	45.00(18.00, 84.00)	0.097
PPCs, n(%)				0.813
No	690 (88.3%)	204 (87.9%)	486 (88.5%)	
Yes	91 (11.7%)	28 (12.1%)	63 (11.5%)	
Gender, n(%)				0.848
Female	131 (16.8%)	38 (16.4%)	93 (16.9%)	
Male	650 (83.2%)	194 (83.6%)	456 (83.1%)	
Preoperative Nutritional Intervention, n(%)				0.279
No	760 (97.3%)	228 (98.3%)	532 (96.9%)	
Yes	21 (2.7%)	4 (1.7%)	17 (3.1%)	
Symptoms, n(%)				0.610
No	11 (1.4%)	2 (0.9%)	9 (1.6%)	
Yes	770 (98.6%)	230 (99.1%)	540 (98.4%)	
Hypertension, n(%)				0.859
No	579 (74.1%)	171 (73.7%)	408 (74.3%)	
Yes	202 (25.9%)	61 (26.3%)	141 (25.7%)	
Diabetes, n(%)				0.596
No	693 (88.7%)	208 (89.7%)	485 (88.3%)	
Yes	88 (11.3%)	24 (10.3%)	64 (11.7%)	
Coronary Heart Disease, n(%)				0.297
No	737 (94.4%)	222 (95.7%)	515 (93.8%)	
Yes	44 (5.6%)	10 (4.3%)	34 (6.2%)	
Surgical History, n(%)				0.653
No	594 (76.1%)	174 (75.0%)	420 (76.5%)	
Yes	187 (23.9%)	58 (25.0%)	129 (23.5%)	
Alcohol History, n(%)				0.731
No	273 (35.0%)	79 (34.1%)	194 (35.3%)	
Yes	508 (65.0%)	153 (65.9%)	355 (64.7%)	
Family History Of Cancer, n(%)				0.106
No	719 (92.1%)	208 (89.7%)	511 (93.1%)	
Yes	62 (7.9%)	24 (10.3%)	38 (6.9%)	
KPS Score, n(%)				0.899
≥ 80	635 (81.3%)	188 (81.0%)	447 (81.4%)	
< 80	146 (18.7%)	44 (19.0%)	102 (18.6%)	
Preoperative Chemotderapy, n(%)				0.600
No	761 (97.4%)	225 (97.0%)	536 (97.6%)	
Yes	20 (2.6%)	7 (3.0%)	13 (2.4%)	
Surgical Procedure, n(%)				0.646
Endoscopic Surgery	260 (33.3%)	80 (34.5%)	180 (32.8%)	
Open Surgery	521 (66.7%)	152 (65.5%)	369 (67.2%)	
Surgical Approach, n(%)				0.985
Left Thoracic Approach	576 (73.8%)	171 (73.7%)	405 (73.8%)	
Right Thoracic Approach	205 (26.2%)	61 (26.3%)	144 (26.2%)	
Anastomosis Technique, n(%)				1.000
Mechanical Anastomosis	778 (99.6%)	231 (99.6%)	547 (99.6%)	
Manual Anastomosis	3 (0.4%)	1 (0.4%)	2 (0.4%)	
Anastomosis Site, n(%)				0.760
Intratdoracic	628 (80.4%)	185 (79.7%)	443 (80.7%)	
Neck	153 (19.6%)	47 (20.3%)	106 (19.3%)	
Elevation Approach, n(%)				0.117
Esophageal Bed	719 (92.1%)	219 (94.4%)	500 (91.1%)	
Retrosternal	62 (7.9%)	13 (5.6%)	49 (8.9%)	
Enteral Nutrition Route, n(%)				0.707
Non	52 (6.7%)	16 (6.9%)	36 (6.6%)	
Nasojejunal tube	680 (87.1%)	204 (87.9%)	476 (86.7%)	
Jejunostomy	49 (6.3%)	12 (5.2%)	37 (6.7%)	
Extent Of Lymph Node Dissection, n(%)				0.931
Standard Two Field	500 (64.0%)	148 (63.8%)	352 (64.1%)	
Above	281 (36.0%)	84 (36.2%)	197 (35.9%)	
Thoracic Adhesions, n(%)				0.977
No	501 (64.1%)	149 (64.2%)	352 (64.1%)	
Yes	280 (35.9%)	83 (35.8%)	197 (35.9%)	
Pleural Effusion, n(%)				0.617
No	687 (88.0%)	202 (87.1%)	485 (88.3%)	
Yes	94 (12.0%)	30 (12.9%)	64 (11.7%)	
Ascites, n(%)				0.740
No	682 (87.3%)	204 (87.9%)	478 (87.1%)	
Yes	99 (12.7%)	28 (12.1%)	71 (12.9%)	
Pleural Metastasis, n(%)				1.000
No	773 (99.0%)	230 (99.1%)	543 (98.9%)	
Yes	8 (1.0%)	2 (0.9%)	6 (1.1%)	
Wide Excision, n(%)				0.979
No	764 (97.8%)	227 (97.8%)	537 (97.8%)	
Yes	17 (2.2%)	5 (2.2%)	12 (2.2%)	
Pain Relief Pump, n(%)				0.390
No	64 (8.2%)	16 (6.9%)	48 (8.7%)	
Yes	717 (91.8%)	216 (93.1%)	501 (91.3%)	
Transfer To ICU, n(%)				1.000
No	766 (98.1%)	228 (98.3%)	538 (98.0%)	
Yes	15 (1.9%)	4 (1.7%)	11 (2.0%)	
Anastomotic Leakage, n(%)				0.979
No	764 (97.8%)	227 (97.8%)	537 (97.8%)	
Yes	17 (2.2%)	5 (2.2%)	12 (2.2%)	
Degree of Tumor Resection, n(%)				1.000
R0	774 (99.1%)	230 (99.1%)	544 (99.1%)	
R1+R2	7 (0.9%)	2 (0.9%)	5 (0.9%)	
Tumor Location, n(%)				0.071
Upper	45 (5.8%)	20 (8.6%)	25 (4.6%)	
Middle	311 (39.8%)	93 (40.1%)	218 (39.7%)	
Lower	425 (54.4%)	119 (51.3%)	306 (55.7%)	
T Stage, n(%)				0.976
T1-T2	336 (43.0%)	100 (43.1%)	236 (43.0%)	
T3-T4	445 (57.0%)	132 (56.9%)	313 (57.0%)	
N Stage, n(%)				0.904
N0	383 (49.0%)	113 (48.7%)	270 (49.2%)	
N+	398 (51.0%)	119 (51.3%)	279 (50.8%)	
M Stage, n(%)				0.988
M0	776 (99.4%)	230 (99.1%)	546 (99.5%)	
M1	5 (0.6%)	2 (0.9%)	3 (0.5%)	
G Stage, n(%)				0.334
G1-G2	428 (54.8%)	121 (52.2%)	307 (55.9%)	
G3-G4	353 (45.2%)	111 (47.8%)	242 (44.1%)	
P Stage, n(%)				0.664
I-II	398 (51.0%)	121 (52.2%)	277 (50.5%)	
III-IV	383 (49.0%)	111 (47.8%)	272 (49.5%)	
Circumferential Cut Edge, n(%)				1.000
>1mm	775 (99.2%)	230 (99.1%)	545 (99.3%)	
≤1mm	6 (0.8%)	2 (0.9%)	4 (0.7%)	
Patdological Tissue Type, n(%)				0.815
Squamous cell carcinoma	518 (66.3%)	154 (66.4%)	364 (66.3%)	
Adenocarcinoma	212 (27.1%)	61 (26.3%)	151 (27.5%)	
Otder	51 (6.5%)	17 (7.3%)	34 (6.2%)	
Lymphovascular Invasion, n(%)				0.817
No	580 (74.3%)	171 (73.7%)	409 (74.5%)	
Yes	201 (25.7%)	61 (26.3%)	140 (25.5%)	
Lymph Node Metastasis, n(%)				0.944
No	389 (49.8%)	116 (50.0%)	273 (49.7%)	
Yes	392 (50.2%)	116 (50.0%)	276 (50.3%)	

BMI, body mass index; FEV1, forced expiratory volume in the first second; MVV, maximum voluntary ventilation; PNI, prognostic nutritional index; NLR, neutrophil-to-lymphocyte ratio; LMR, lymphocyte-to-monocyte ratio; PLR, platelet-to-lymphocyte ratio; dNLR, derived neutrophil-to-lymphocyte ratio; NLPR, neutrophil-to-lymphocyte*platelet ratio; SIRI, systemic inflammation response index; AISI, aggregate index of systemic inflammation; SII, systemic immune-inflammation index; PIV, pan-immune-inflammation value; LDH, lactate dehydrogenase; 5’-NT, 5’-nucleotidase; ALT, alanine aminotransferase; AST, aspartate aminotransferase; KPS, karnofsky performance status; ICU, intensive care unit.

### 3.2 Identifying risk factors for PPCs using univariate and multivariate logistic regression analysis

Eleven variables were shown to be substantially linked to PPCs in patients with EC in the univariate logistic regression analysis (**Table 3**). Risk factors included neutrophil count, eosinophil count, monocyte count, dNLR, PLR, SII, extended lymph node dissection, intraoperative pleural metastasis detection, postoperative ICU admission, and anastomotic fistula formation; LMR showed protective effects. There was no statistical significance in the remaining clinical and pathological factors.

**Table 3:** Results of single-factor logistic regression.

Variables	Partial regression coefficient	Standard error	*Z-value*	OR(95%CI)	*p-value*
Gender, n(%)					
Female				Reference	
Male	0.377	0.397	0.949	1.457(0.670~3.172)	0.343
Age,Median(Q1,Q3)	0.019	0.017	1.146	1.019(0.987~1.053)	0.252
Preoperative Nutritional Intervention, n(%)					
No				Reference	
Yes	0.522	0.651	0.802	1.686(0.471~6.036)	0.422
Symptoms, n(%)					
No				Reference	
Yes	0.037	1.069	0.035	1.038(0.128~8.437)	0.972
Hypertension, n(%)					
No				Reference	
Yes	-0.113	0.313	0.362	0.893(0.483~1.650)	0.718
Diabetes, n(%)					
No				Reference	
Yes	-0.731	0.535	1.367	0.481(0.169~1.373)	0.172
Coronary Heart Disease, n(%)					
No				Reference	
Yes	-0.765	0.742	1.032	0.465(0.109~1.990)	0.302
Surgical History, n(%)					
No				Reference	
Yes	-0.677	0.375	1.804	0.508(0.244~1.060)	0.071
Smoking Index,Median(Q1,Q3)	0.000	0.000	0.332	1.000(1.000~1.000)	0.740
Alcohol History, n(%)					
No				Reference	
Yes	0.438	0.299	1.465	1.550(0.862~2.786)	0.143
Family History Of Cancer, n(%)					
No				Reference	
Yes	0.168	0.500	0.337	1.183(0.444~3.152)	0.736
BMI,Median(Q1,Q3)	0.025	0.041	0.612	1.026(0.946~1.112)	0.541
Actual FEV1 Value,Median(Q1,Q3)	-0.103	0.249	0.413	0.902(0.553~1.471)	0.680
MVV,Median(Q1,Q3)	-0.006	0.005	1.230	0.994(0.985~1.003)	0.219
MVV Predicted Value,Median(Q1,Q3)	-0.002	0.006	0.271	0.998(0.986~1.010)	0.786
Neutrophils,Median(Q1,Q3)	0.098	0.049	2.010	1.103(1.002~1.213)	0.044
Lymphocyte,Median(Q1,Q3)	-0.308	0.236	1.306	0.735(0.463~1.167)	0.192
Eosinophils,Median(Q1,Q3)	1.805	0.786	2.296	6.083(1.302~28.414)	0.022
Basophil,Median(Q1,Q3)	6.713	4.135	1.623	823.422(0.249~2727029.954)	0.104
Monocyte,Median(Q1,Q3)	1.443	0.626	2.303	4.232(1.240~14.449)	0.021
PNI,Median(Q1,Q3)	-0.041	0.026	1.535	0.960(0.912~1.011)	0.125
NLR,Median(Q1,Q3)	0.043	0.025	1.730	1.044(0.994~1.096)	0.084
LMR,Median(Q1,Q3)	-0.264	0.098	2.697	0.768(0.634~0.930)	0.007
PLR,Median(Q1,Q3)	0.003	0.001	2.443	1.003(1.001~1.006)	0.015
dNLR,Median(Q1,Q3)	0.121	0.058	2.103	1.129(1.008~1.264)	0.035
NLPR,Median(Q1,Q3)	5.290	5.023	1.053	198.413(0.011~3739909.297)	0.292
SIRI,Median(Q1,Q3)	0.034	0.030	1.141	1.035(0.976~1.097)	0.254
AISI,Median(Q1,Q3)	0.000	0.000	1.284	1.000(1.000~1.000)	0.199
SII,Median(Q1,Q3)	0.000	0.000	2.104	1.000(1.000~1.000)	0.035
PIV,Median(Q1,Q3)	0.000	0.000	1.284	1.000(1.000~1.000)	0.199
Albumin-Globulin Ratio,Median(Q1,Q3)	-0.265	0.421	0.628	0.767(0.336~1.752)	0.530
Complemen C1q,Median(Q1,Q3)	-0.001	0.004	0.344	0.999(0.991~1.006)	0.731
LDH,Median(Q1,Q3)	0.001	0.003	0.250	1.001(0.994~1.007)	0.803
5’-NT,Median(Q1,Q3)	0.002	0.080	0.028	1.002(0.857~1.173)	0.978
Total Protein,Median(Q1,Q3)	0.001	0.023	0.023	1.001(0.957~1.046)	0.981
Albumin,Median(Q1,Q3)	-0.036	0.035	1.027	0.964(0.899~1.034)	0.304
Total Bilirubin,Median(Q1,Q3)	-0.000	0.026	0.010	1.000(0.949~1.053)	0.992
ALT,Median(Q1,Q3)	0.006	0.006	0.964	1.006(0.994~1.018)	0.335
AST,Median(Q1,Q3)	0.005	0.004	1.079	1.005(0.996~1.013)	0.281
Urea,Median(Q1,Q3)	0.024	0.087	0.274	1.024(0.863~1.215)	0.784
Creatinine,Median(Q1,Q3)	-0.003	0.009	0.314	0.997(0.979~1.015)	0.754
Uric Acid,Median(Q1,Q3)	0.001	0.002	0.689	1.001(0.998~1.005)	0.491
KPS Score, n(%)					
≥ 80				Reference	
< 80	0.148	0.333	0.446	1.160(0.604~2.226)	0.656
Preoperative Chemotherapy, n(%)					
No				Reference	
Yes	0.348	0.781	0.445	1.416(0.307~6.539)	0.656
Surgery Duration,Median(Q1,Q3)	0.002	0.003	0.903	1.002(0.997~1.008)	0.366
Surgical Procedure, n(%)					
Endoscopic Surgery				Reference	
Open Surgery	0.223	0.295	0.756	1.250(0.701~2.229)	0.449
Surgical Approach, n(%)					
Left Thoracic Approach				Reference	
Right Thoracic Approach	0.551	0.282	1.953	1.735(0.998~3.014)	0.051
Anastomosis Technique, n(%)					
Mechanical Anastomosis				Reference	
Manual Anastomosis	-12.527	624.194	0.020	0.000(0.000~Inf)	0.984
Anastomosis Site, n(%)					
Intrathoracic				Reference	
Neck	0.202	0.324	0.622	1.224(0.648~2.311)	0.534
Elevation Approach, n(%)					
Esophageal Bed				Reference	
Retrosternal	0.279	0.432	0.645	1.321(0.566~3.083)	0.519
Enteral Nutrition Route, n(%)					
Non				Reference	
Nasojejunal tube	0.342	0.620	0.552	1.408(0.418~4.744)	0.581
Jejunostomy	0.756	0.750	1.008	2.129(0.490~9.257)	0.314
Tumor Volume,Median(Q1,Q3)	-0.000	0.001	0.268	1.000(0.998~1.002)	0.789
Extent Of Lymph Node Dissection, n(%)					
Standard Two Field				Reference	
Above	0.552	0.270	2.046	1.737(1.023~2.946)	0.041
Thoracic Adhesions, n(%)					
No				Reference	
Yes	0.031	0.278	0.110	1.031(0.598~1.779)	0.913
Pleural Effusion, n(%)					
No				Reference	
Yes	-1.055	0.607	1.737	0.348(0.106~1.145)	0.082
Ascites, n(%)					
No				Reference	
Yes	-0.024	0.402	0.059	0.977(0.444~2.146)	0.953
Pleural Metastasis, n(%)					
No				Reference	
Yes	2.086	0.828	2.519	8.050(1.589~40.786)	0.012
Wide Excision, n(%)					
No				Reference	
Yes	0.445	0.786	0.566	1.561(0.334~7.290)	0.571
Pain Relief Pump, n(%)					
No				Reference	
Yes	1.893	1.020	1.856	6.638(0.900~48.970)	0.063
Transfer To ICU, n(%)					
No				Reference	
Yes	2.315	0.622	3.725	10.126(2.995~34.237)	0.000
Anastomotic Leakage, n(%)					
No				Reference	
Yes	2.864	0.629	4.555	17.527(5.112~60.099)	0.000
Degree of Tumor Resection, n(%)					
R0				Reference	
R1+R2	0.665	1.126	0.590	1.944(0.214~17.668)	0.555
Tumor Location, n(%)					
Upper				Reference	
Middle	0.118	0.647	0.182	1.125(0.317~3.999)	0.855
Lower	-0.190	0.644	0.296	0.827(0.234~2.921)	0.768
T Stage, n(%)					
T1-T2				Reference	
T3-T4	-0.067	0.270	0.248	0.935(0.551~1.586)	0.804
N Stage, n(%)					
N0				Reference	
N+	0.215	0.269	0.798	1.240(0.731~2.102)	0.425
M Stage, n(%)					
M0				Reference	
M1	-13.529	840.274	0.016	0.000(0.000~Inf)	0.987
G Stage, n(%)					
G1-G2				Reference	
G3-G4	-0.433	0.280	1.548	0.649(0.375~1.122)	0.122
P Stage, n(%)					
I-II				Reference	
III-IV	0.200	0.269	0.746	1.222(0.722~2.068)	0.456
Circumferential Cut Edge, n(%)					
>1mm				Reference	
≤1mm	0.954	1.163	0.821	2.597(0.266~25.352)	0.412
Pathological Tissue Type, n(%)					
Squamous cell carcinoma				Reference	
Adenocarcinoma	-0.246	0.315	0.781	0.782(0.422~1.450)	0.435
Other	-0.377	0.625	0.603	0.686(0.201~2.336)	0.547
Lymphovascular Invasion, n(%)					
No				Reference	
Yes	-0.006	0.308	0.020	0.994(0.544~1.816)	0.984
Lymph Node Metastasis, n(%)					
No				Reference	
Yes	0.240	0.269	0.890	1.271(0.750~2.154)	0.374

BMI, body mass index; FEV1, forced expiratory volume in the first second; MVV, maximum voluntary ventilation; PNI, prognostic nutritional index; NLR, neutrophil-to-lymphocyte ratio; LMR, lymphocyte-to-monocyte ratio; PLR, platelet-to-lymphocyte ratio; dNLR, derived neutrophil-to-lymphocyte ratio; NLPR, neutrophil-to-lymphocyte*platelet ratio; SIRI, systemic inflammation response index; AISI, aggregate index of systemic inflammation; SII, systemic immune-inflammation index; PIV, pan-immune-inflammation value; LDH, lactate dehydrogenase; 5’-NT, 5’-nucleotidase; ALT, alanine aminotransferase; AST, aspartate aminotransferase; KPS, karnofsky performance status; ICU, intensive care unit.

**Figure 2:**
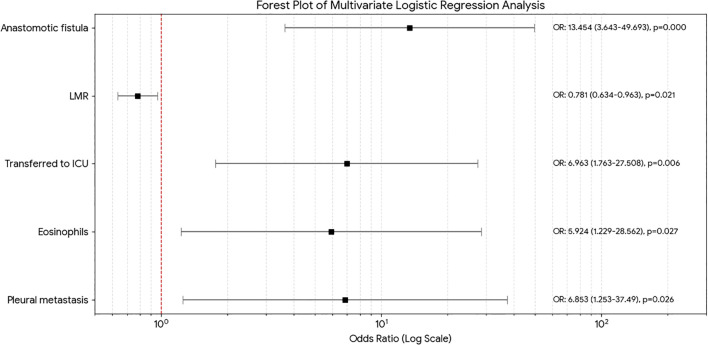
Forest plot of risk factors. LMR, lymphocyte-to-monocyte ratio; ICU, intensive care unit.

Five independent predictors of PPCs were finally found using forward stepwise multiple logistic regression analysis. The eosinophil count (OR = 5.924, p=0.027), intraoperative pleural metastasis (OR = 6.853, p=0.026), postoperative ICU admission(OR = 6.963, p=0.006), and postoperative anastomotic leakage (OR = 13.454, p=0.000) were found to be significant risk factors, while the LMR (OR = 0.791, p=0.021) was a protective factor. The five independent predictors found by the multivariate logistic regression are shown graphically in **Figure 2**’s forest plot. **Table 4** displays the multivariate logistic regression analysis’s findings.

**Table 4:** Multivariate logistic regression results.

Variables	Partial regression coefficient	Standard error	*Z-value*	OR(95%CI)	*p-value*
(Intercept)	-1.658	0.403	4.116	0.191(0.087~0.420)	0.000
Eosinophils,Median(Q1,Q3)	1.779	0.803	2.216	5.924(1.229~28.562)	0.027
LMR,Median(Q1,Q3)	-0.247	0.107	2.310	0.781(0.634~0.963)	0.021
Pleural Metastasis, n(%)					
No				Reference	
Yes	1.925	0.867	2.220	6.853(1.253~37.490)	0.026
Transfer To ICU, n(%)					
No				Reference	
Yes	1.941	0.701	2.769	6.963(1.763~27.508)	0.006
Anastomotic Leakage, n(%)					
No				Reference	
Yes	2.599	0.667	3.899	13.454(3.643~49.693)	0.000

LMR, lymphocyte-to-monocyte ratio; ICU, intensive care unit.

### 3.3 Construction of nomograms

**Figure 3:**
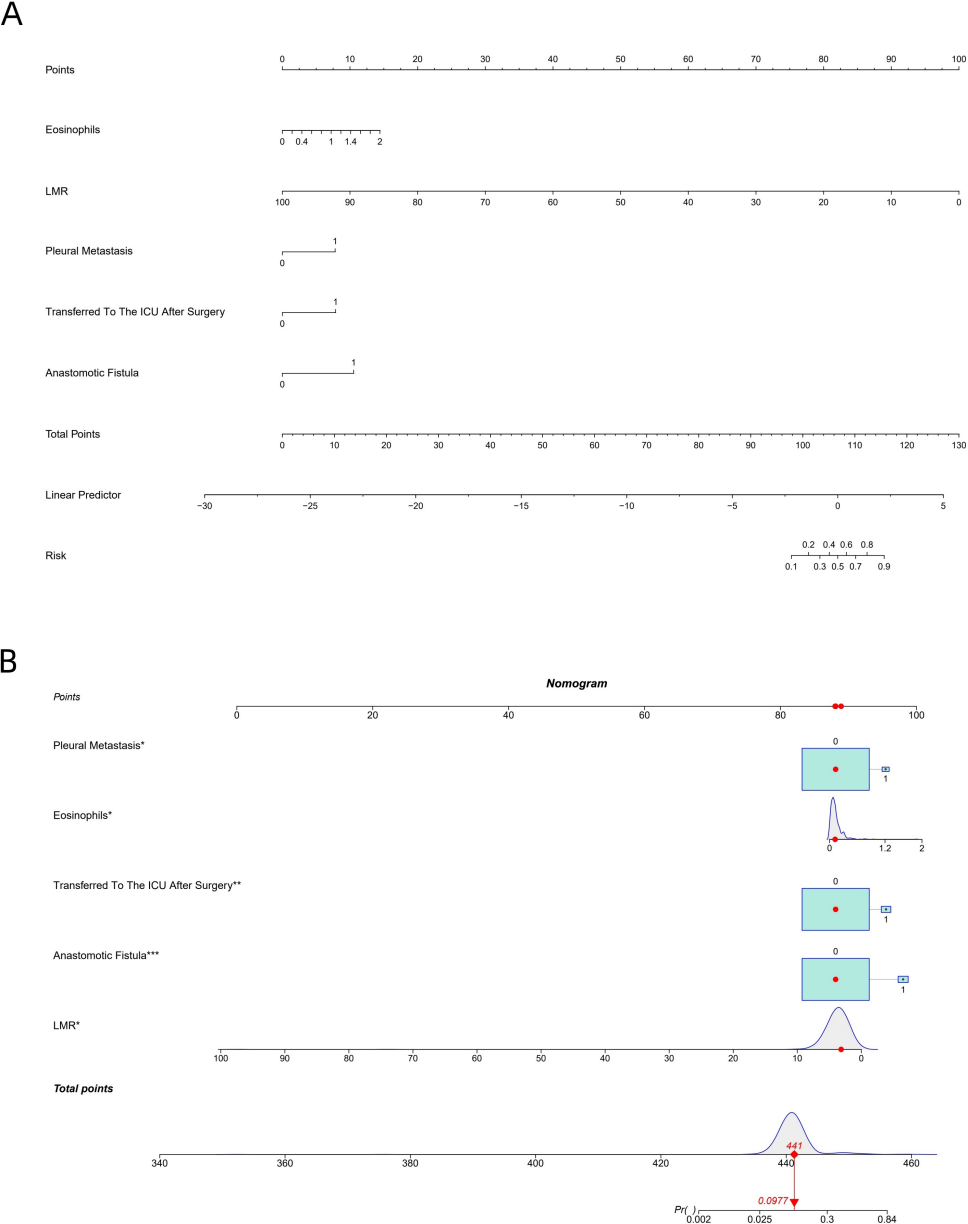
**(A, B)** Nomogram for predicting PPCs. LMR, lymphocyte-to-monocyte ratio.

Based on the five independent factors found in the initial multivariable analysis, a nomogram was created for exploratory and illustrative reasons. The purpose of this visualization tool is to show how these elements work together, but it is not meant to be the main prediction model used in clinical settings. Among these, anastomotic fistula, postoperative ICU admission and pleural metastases are binary variables (0 denotes absence, 1 denotes presence). R statistical software was used to create a nomogram for PPCs in patients with EC based on the aforementioned algorithm. A simple nomogram is shown in **Figure 3A**, and a stylized nomogram is shown in **Figure 3B**.

Additionally, we performed multicollinearity diagnostics (**Table 5**). All of the variables in the final model had variance inflation factors (VIFs) of less than 1.02, which guaranteed the stability of the model’s findings and showed no substantial multicollinearity among the variables.

**Table 5:** Collinearity diagnosis results.

Variable	VIF
Transferred To The ICU After Surgery	1.006624
Eosinophils	1.007819
LMR	1.005374
Pleural Metastasis	1.010970
Anastomotic Leakage	1.005670

LMR, lymphocyte-to-monocyte ratio; VIF, variance inflation factor.

### 3.4 Model performance and validation

**Figure 4:**
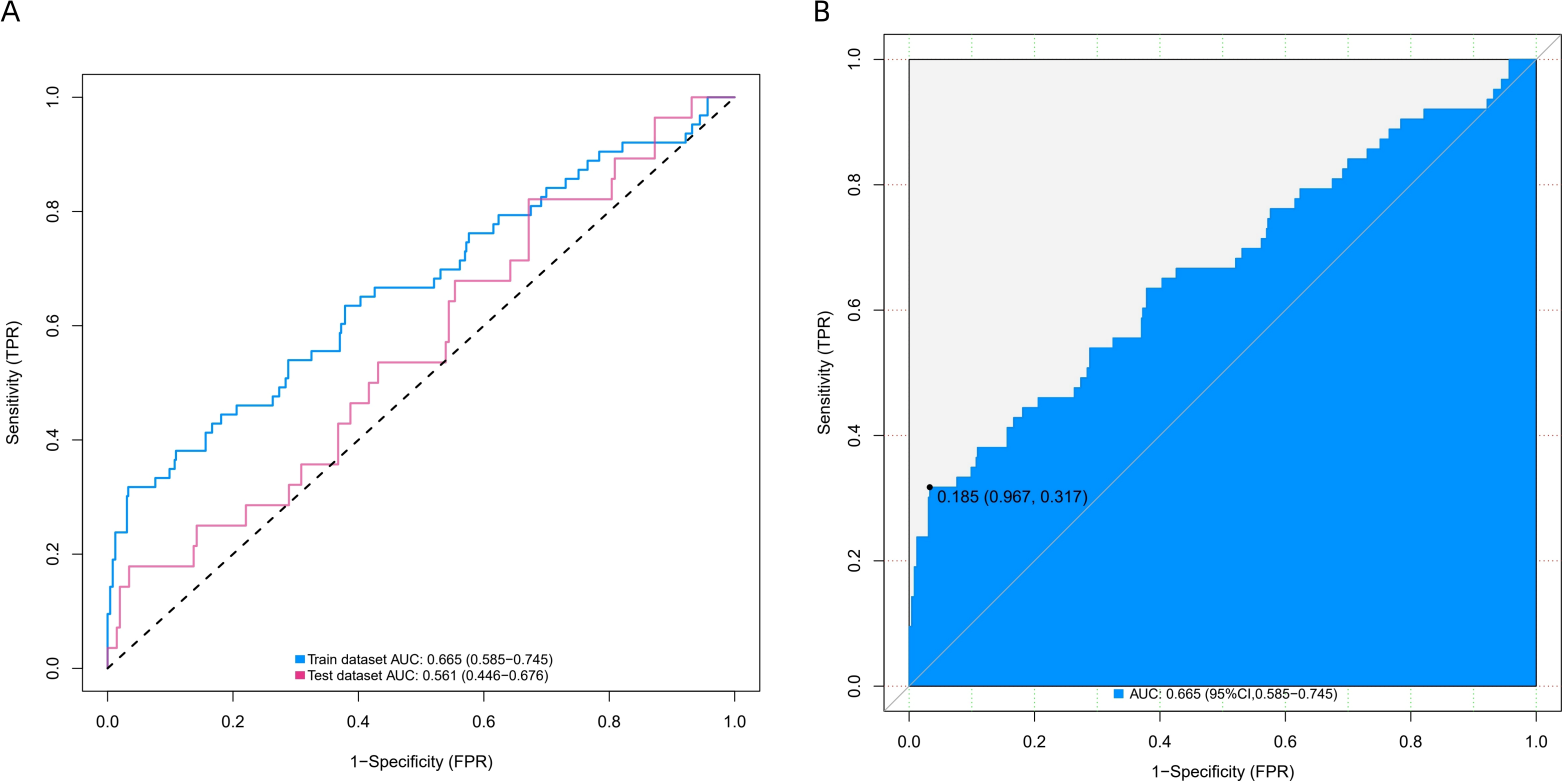
**(A, B)** ROC curve results. ROC, receiver operating characteristic; AUC, area under the curve.

**Figure 5:**
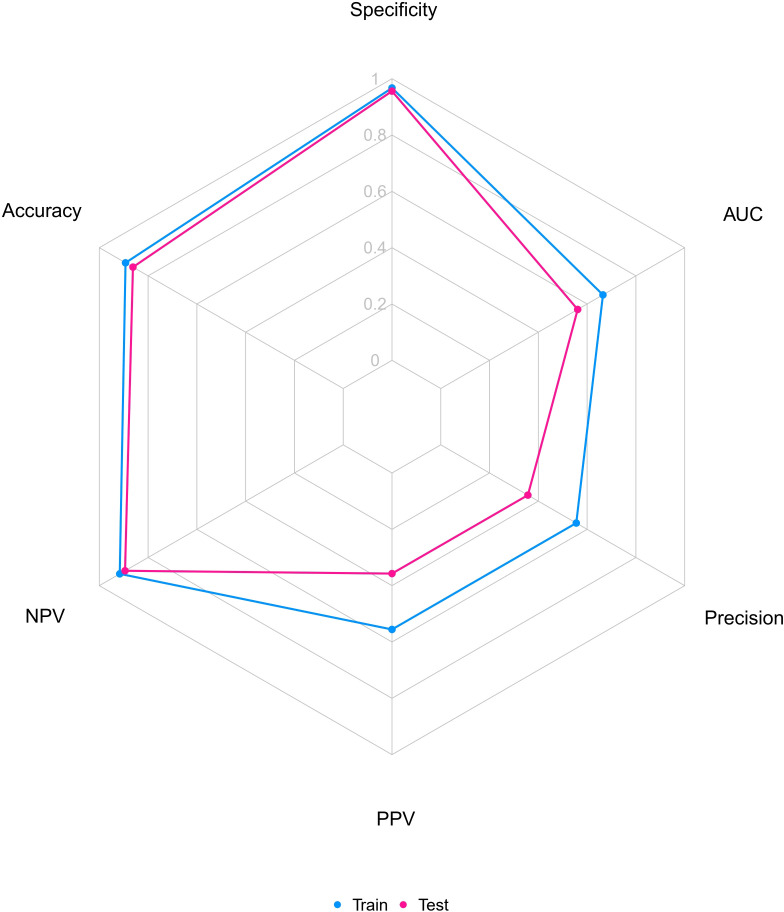
Radar chart of the prediction model. NPV, negative predictive value; AUC, area under the curve.

To evaluate the explanatory power and robustness of the identified risk factors, this study uses ROC curves, bias corrected C-index and radar charts to assess the predictive model’s performance. **Figure 4A** shows the ROC curve. The model exhibits moderate but stable discriminatory capability, as shown by the blue curve representing the training set (AUC = 0.665, 95% CI 0.585–0.745) and the pink curve representing the test set (AUC = 0.561, 95% CI 0.446–0.676). The model shows consistent performance across datasets, despite the AUC values reflecting the inherent difficulties in predicting PPCs simply based on preoperative and intraoperative parameters. The predictive model reaches its ideal cutoff value when specificity is 0.967 and sensitivity is 0.317 on the training set, as **Figure 4B** illustrates. The Youden index at this point is 0.185. The detailed results of the ROC curve are listed in **Table 6** for reference. We used the Bootstrap approach (with 1000 resamples) to compute the bias corrected C-index. The model obtained a bias corrected C-index of 0.666 on the training set and 0.557 on the independent validation set, according to the results. A radar chart showing the model’s overall performance across several metrics on the training and test datasets is shown in **Figure 5**. The model retains strong and consistent classification capabilities across many datasets with no indications of overfitting, as shown by the remarkably identical graphical profiles covering large areas.

**Table 6:** Detailed results of ROC analysis.

Characteristics	Training set	Validation set
Threshold	0.185	0.185
Specificity	0.967	0.956
Sensitivity	0.317	0.179
Accuracy	0.893	0.862
TN	470.000	195.000
TP	20.000	5.000
FN	43.000	23.000
FP	16.000	9.000
NPV	0.916	0.894
PPV	0.556	0.357
FDR	0.444	0.643
FPR	0.033	0.044
TPR	0.317	0.179
TNR	0.967	0.956
FNR	0.683	0.821
1-Specificity	0.033	0.044
1-Sensitivity	0.683	0.821
1-Accuracy	0.107	0.138
1-NPV	0.084	0.106
1-PPV	0.444	0.643
Precision	0.556	0.357
Recall	0.317	0.179
Youden	0.285	0.134
Closest.topleft	0.467	0.677
AUC	0.665(0.585-0.745)	0.561(0.446-0.676)
z	4.041	1.043
p	<0.001	0.297

TN, true negative; TP, true positive; FN, false negative; FP, false positive; NPV, negative predictive value; PPV, positive predictive value; FDR, false discovery rate; FPR, false positive rate; TPR, true positive rate; TNR, true negative rate; FNR, false negative rate; AUC, area under the curve; Youden, youden’s index; Closest.topleft, closest to top-left corner criterion.

**Figure 6:**
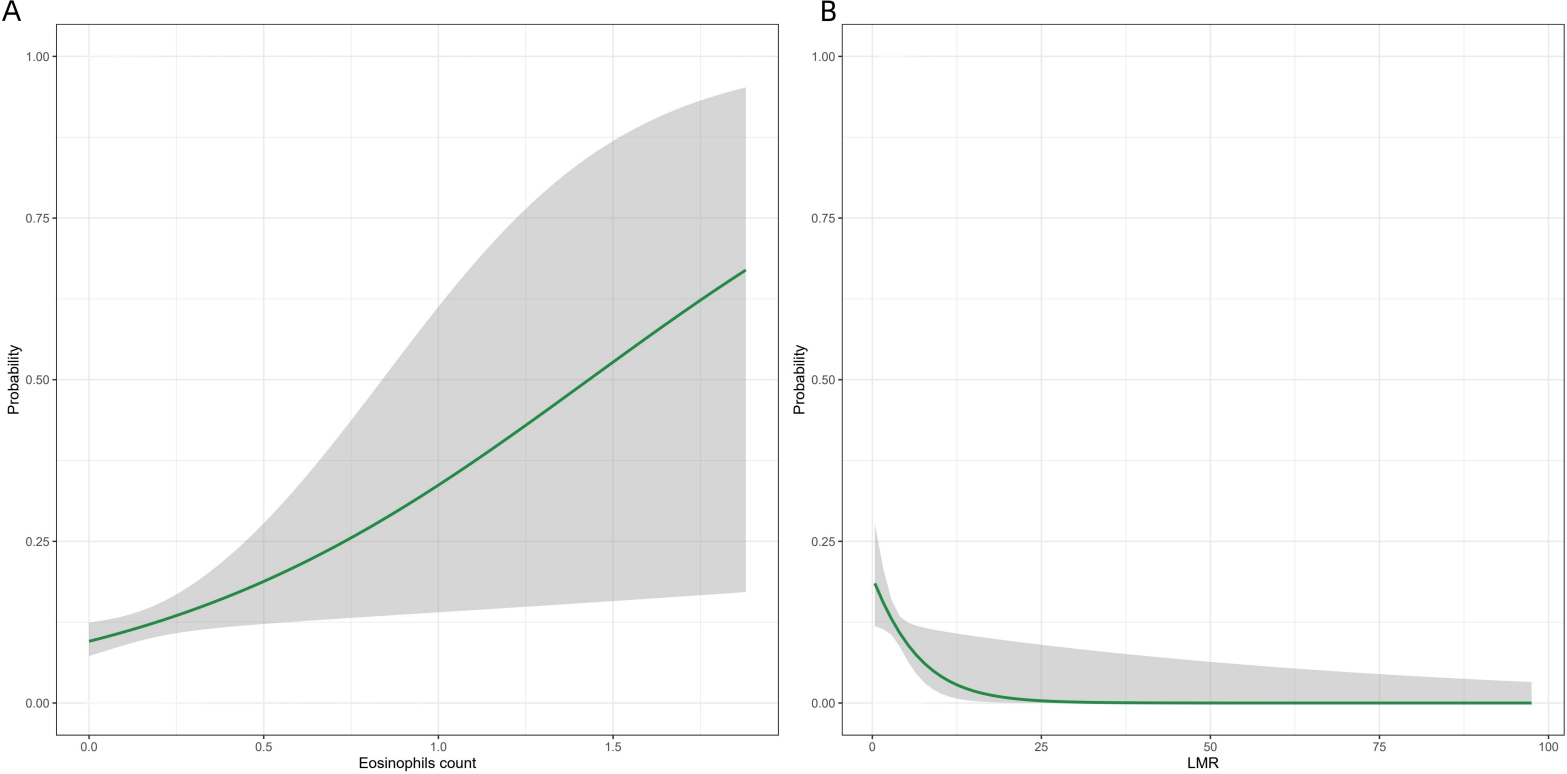
**(A, B)** Calibration curve of the predictive model.

The model shows satisfactory calibration following correction, as seen in **Figure 6**, where the calibrated curve nearly resembles the ideal diagonal line. We used the Hosmer-Lemeshow goodness-of-fit test. The test did not produce any statistically significant results in either the validation set (p = 0.628) or the training set (p = 0.371). This shows that, over the whole risk spectrum, the estimated risks derived from these inflammatory and surgical factors show great consistency with actual observed probabilities, demonstrating that the model accurately reflects the correlation between the identified factors and clinical outcomes.

**Figure 7:**
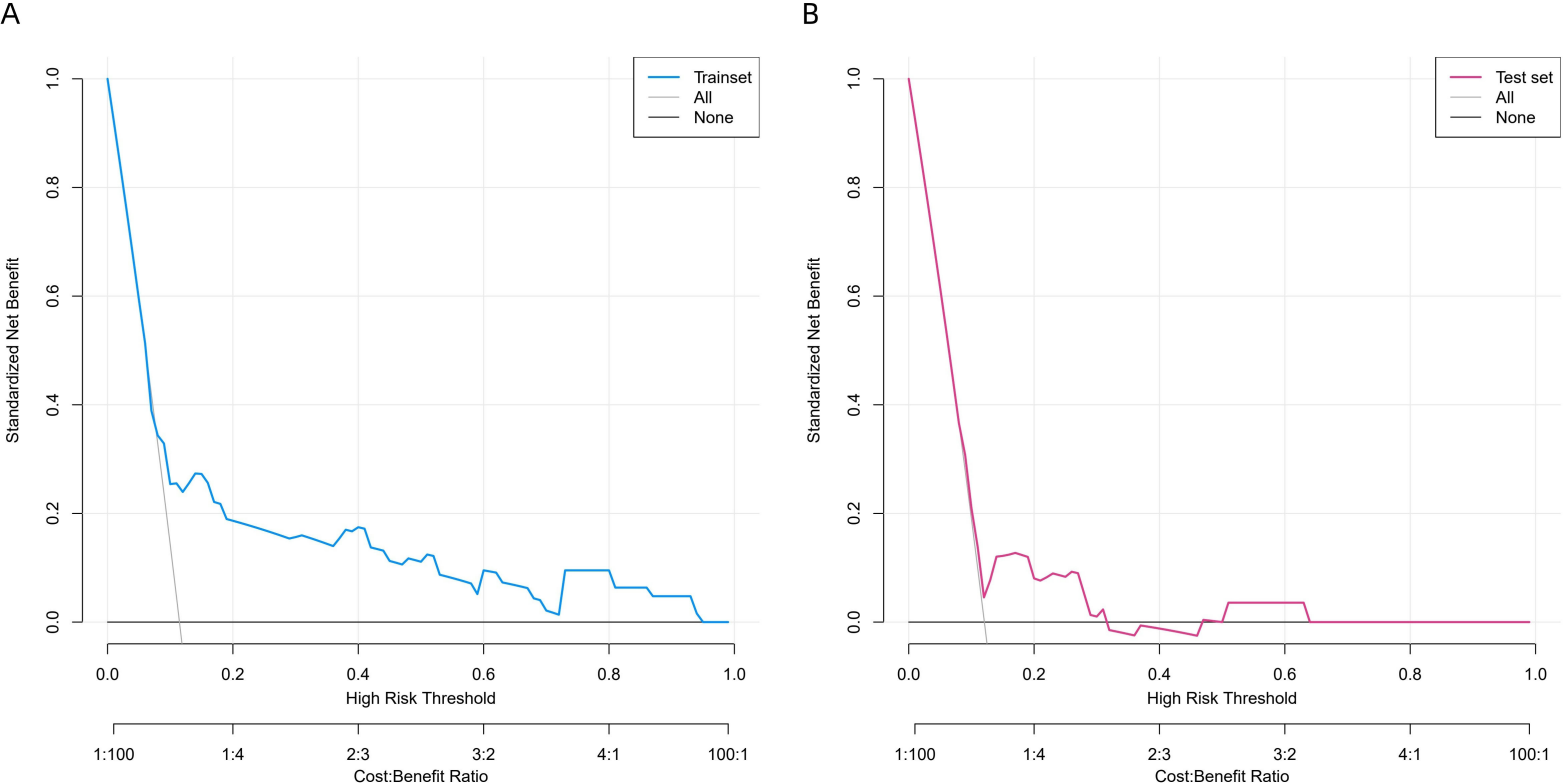
**(A, B)** DCA of the training set and validation set. DCA, decision curve analysis.

**Figure 8:**
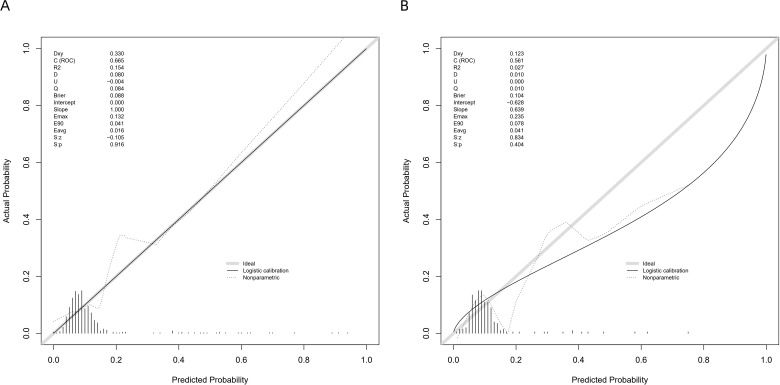
**(A, B)** CIC of the training set and validation set. CIC, clinical impact curves.

This study used decision curve analysis (DCA) and clinical impact curves (CIC) to assess the clinical relevance of the risk model. In the DCA of the training set (**Figure 7A**) and validation set (**Figure 7B**), the model’s net benefit curve showed positive clinical net benefit throughout a wide range of threshold probabilities (about 0.1 to 0.8). Both the number of people the model identified as high risk and the number of people who experienced actual events show a steady decline as the high-risk threshold varies in the CIC of **Figures 8A, B**. The proximity of the curves validates that incorporating Eosinophils, LMR, Intraoperative Pleural Metastasis, Postoperative ICU Admission and Anastomotic Leakage into risk stratification offers tangible clinical value, allowing clinicians to identify vulnerable patients who may benefit from intensified perioperative management.

### 3.5 Exploratory assessment of variable contributions

**Figure 9:**
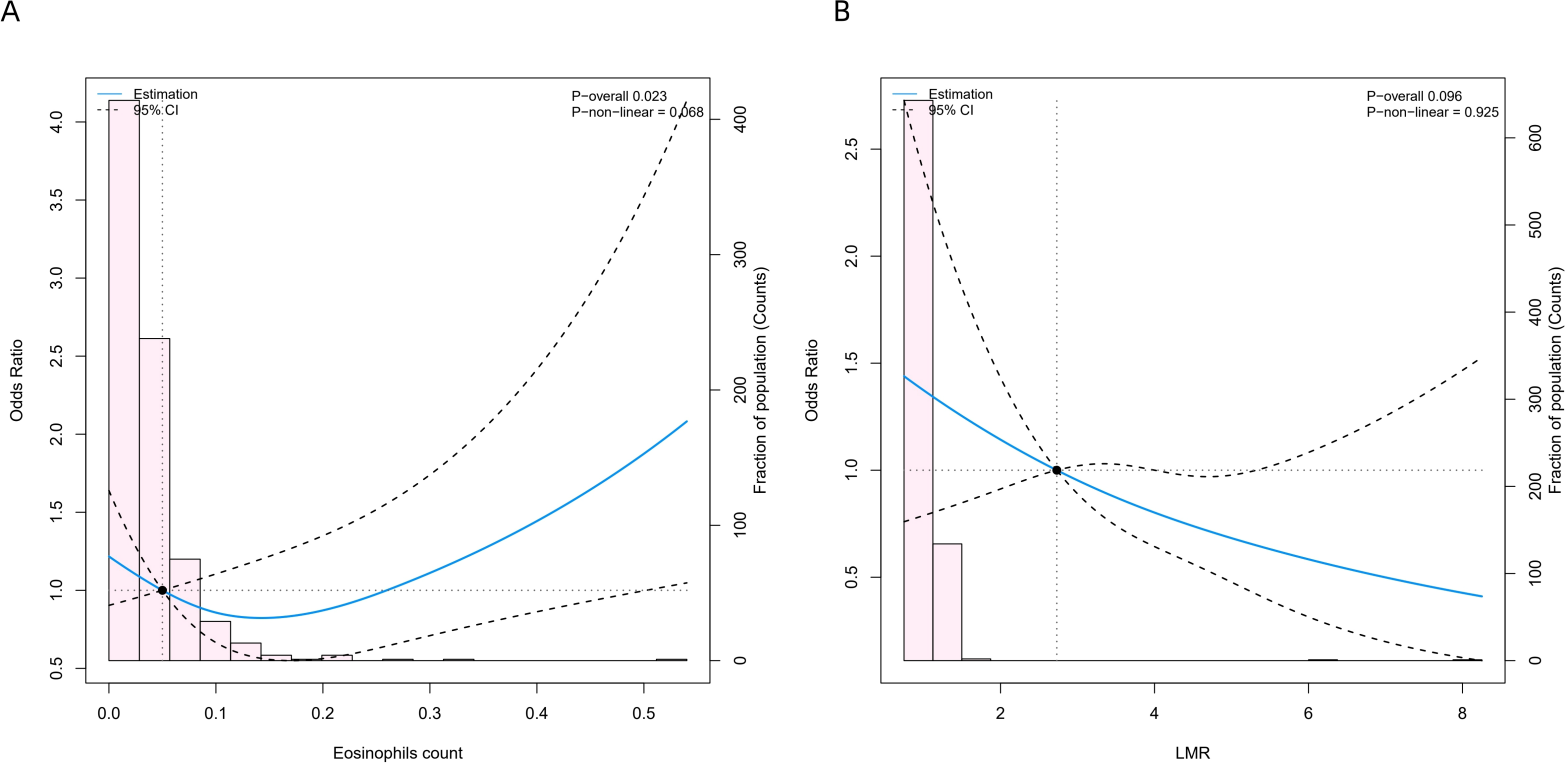
**(A, B)** The SHAP analysis variable importance plot. SHAP, shapley additive explanations; LMR, lymphocyte-to-monocyte ratio.

The SHAP analysis variable importance plot in **Figure 9** illustrates the magnitude of association between each risk factor and PPCs within the multivariable risk analysis. The five risk factors’ relative contributions to the risk probability are reflected in the SHAP values displayed in the figure. The most important predictor, according to analysis, is “LMR”. “Eosinophils count”, “Anastomotic Leakage”, “Postoperative ICU Admission” and “Pleural Metastasis” come next. The inflammatory marker “LMR” showed a notable protective effect among these, while “Eosinophils count” demonstrated a strong positive association with increased risk.

**Figure 10:**
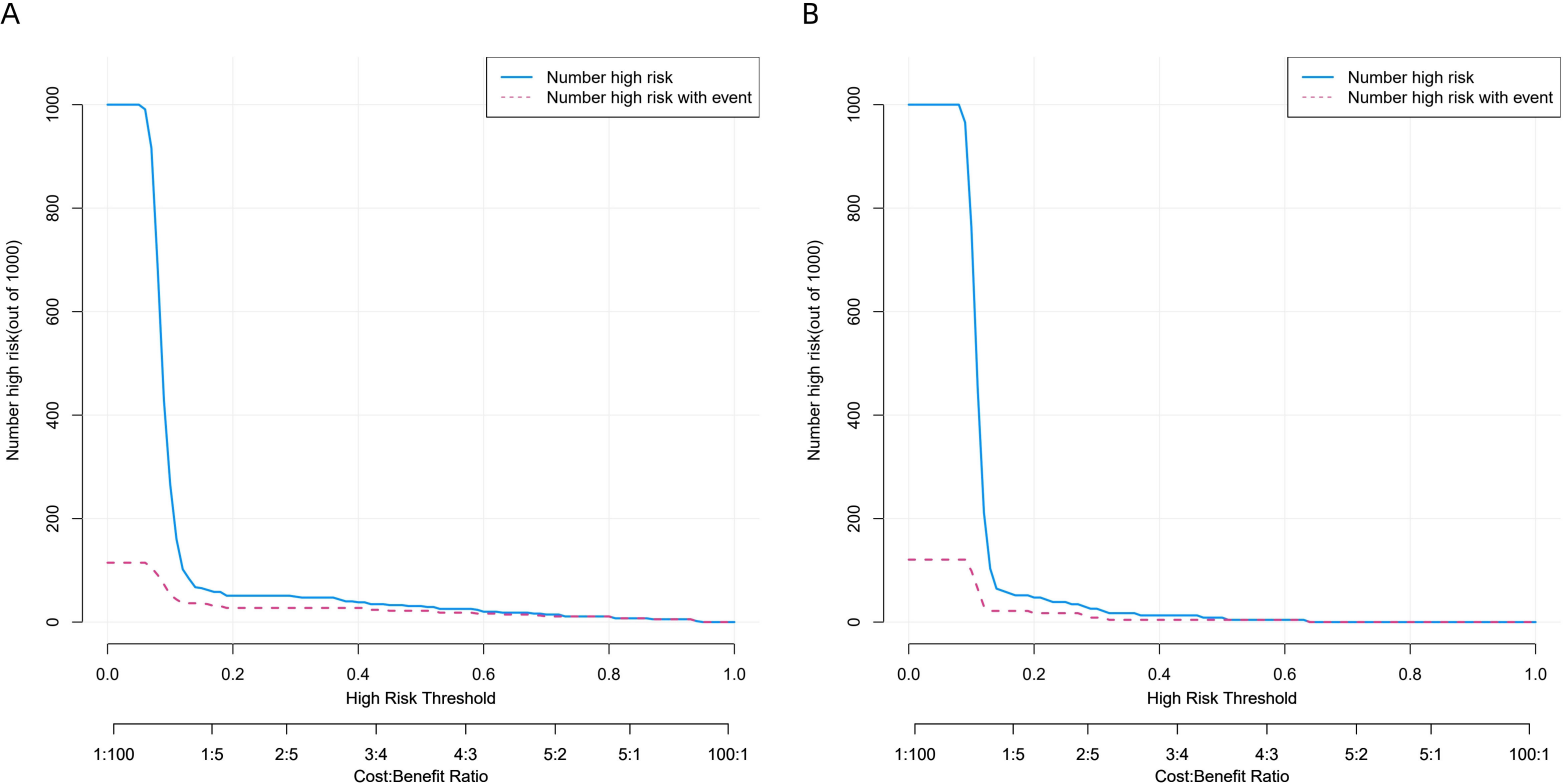
**(A, B)** The SHAP dependency plot. SHAP, shapley additive explanations; LMR, lymphocyte-to-monocyte ratio.

The SHAP dependency plot, which consists of five subplots examining five different indicators, is shown in **Figure 10**. The horizontal axis in each subplot represents the value range of the related indicator, and the vertical axis is the SHAP values. The biological and clinical relationships between risk factors and SHAP values are revealed using data distribution visualization, which makes it evident how each indicator modulates the risk of PPCs across a range of values. More significantly, it shows that the model views these inflammatory markers and surgical complications as interacting and amplifying one another rather than acting independently, ultimately determining the overall clinical risk profile.

### 3.6 Logistic curve

**Figure 11:**
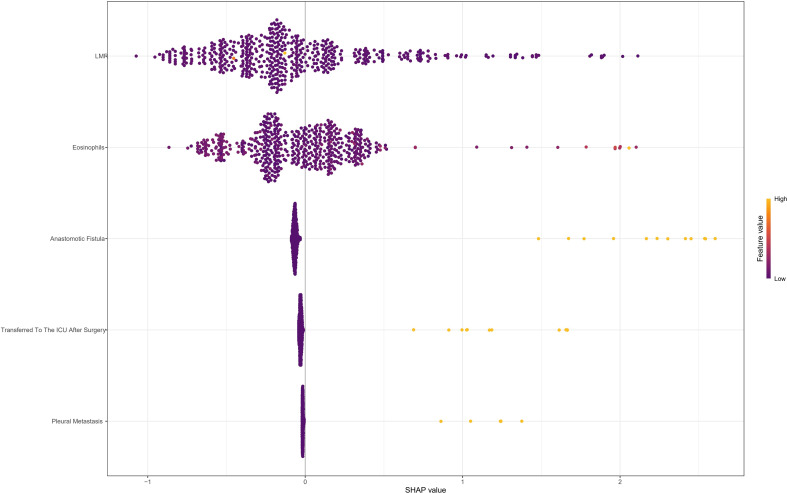
The logistic curves for three continuous variables. LMR, lymphocyte-to-monocyte ratio.

The logistic curves for three continuous variables— eosinophils count, and LMR—are shown in (**Figures 11A, B**). In keeping with the typical features of a logistic function, these curves consistently show a large nonlinear effect of each variable on the response probability. They make evident the patterns of correlation between many factors and the likelihood of PPCs occurring.

### 3.7 RCS curve

**Figure 12:**
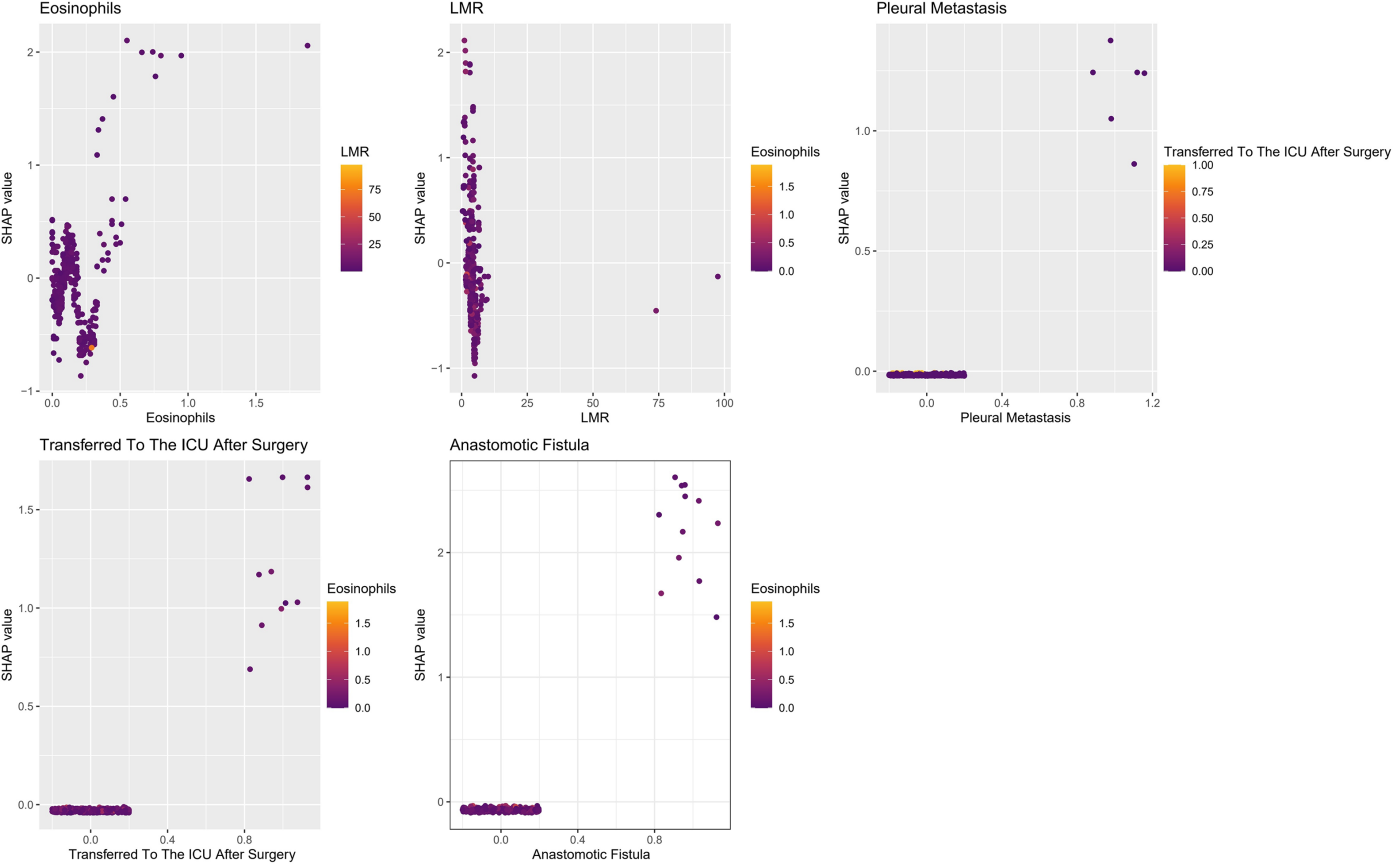
RCS curves for three continuous variables. LMR, lymphocyte-to-monocyte ratio.

To examine the relationship between continuous variables and PPCs, (**Figures 12A, B**) shows the RCS curves for eosinophil count and LMR, respectively. The findings show that eosinophil count show significant overall associations with outcomes (overall P < 0.05). There is no evidence of significant nonlinear trends (P−non−linear > 0.05), indicating effects closer to a linear pattern. However, the association between LMR and PPCs was not statistically significant (P−overall = 0.096, P−non−linear = 0.925).

## 4 Discussion

The overall incidence of PPCs in our study was determined to be 11.65%. Patients’ quick recovery and long-term survival are significantly hampered by PPCs (40, 41). Additionally, they greatly extend hospital stays, which puts a strain on healthcare resources and increases the financial burden on patients’ families (42–44). The postoperative ICU admission, eosinophil count, LMR, intraoperative pleural metastasis, and postoperative anastomotic leakage are the five major independent risk factors identified in this comprehensive risk factor analysis. This method makes it possible to identify high-risk individuals based on biological and surgical characteristics, giving medical professionals vital information for targeted therapeutic interventions and focused prevention during the perioperative phase.

Transfer To ICU, intraoperative pleural metastasis and postoperative anastomotic leakage are the strongest independent risk variables (OR > 6) for predicting PPCs, as this study clearly demonstrates. Our results are in line with the study by Jin et al., which found anastomotic leaking to be an independent risk factor for postoperative pneumonia (19). Nevertheless, neither intraoperative pleural metastasis or postoperative ICU admission nor the connection between the three risk factors were included in their study. This clearly identifies a group of patients who are at high risk. When patients have intraoperative evidence of pleural metastasis, clinicians must take all essential precautions to prevent anastomotic leakage. Patients with anastomotic leakage complicated by pleural metastases need to be monitored at the highest level due to the compounding severity of their situation.

Preoperative peripheral blood eosinophil levels were found to be an independent strong risk factor (OR = 6.499), which is an important discovery. This contradicts the conventional understanding that eosinophils are only involved in parasitic or allergic illnesses. To the best of our knowledge, this is the first study to show a correlation between eosinophil counts and PPCs following surgery in patients with EC, as well as the first to integrate this discovery into a prediction model. We speculate that patients may have a Th2-dominant immune response as a baseline condition if preoperative high eosinophil counts are present (45–47). Postoperative surgical trauma may intensify this particular immunological microenvironment. On the one hand, it releases harmful granule contents such major basic protein and eosinophil cationic protein, which directly harm the airway epithelium (48–51). On the other hand, the Th1/cytotoxic immune response, which is crucial for fighting bacterial infections, may be weakened by Th2 dominance, greatly raising the risk of postoperative pneumonia (52–54). Eosinophils might therefore be thought of as a new, easily accessible biomarker that reflects certain immunological vulnerabilities in the body.

In contrast to eosinophils, the LMR demonstrated a protective effect in this study. A reduction in lymphocyte-mediated antitumor immune function and an increase in monocyte-mediated systemic inflammation are typical of LMR composite indicator (55). Our results show that a higher preoperative LMR is consistently associated with a lower risk of PPCs. This is consistent with its value in long-term prognosis studies across several cancers (56–60). This highlights the importance of maintaining a balance between the patient’s preoperative immune function and inflammatory levels for a smooth perioperative period. It also provides theoretical support for lowering complication risks by enhancing the patient’s baseline immune status.

This study’s creativity and methodical approach are its main advantages. This is the first time that a number of novel systemic inflammatory markers have been systematically evaluated and integrated into a risk analysis framework for PPCs following EC surgery. The independent predictive value of eosinophils and LMR was successfully validated. This gives the field a new biological dimension and sheds light on the immunological and inflammatory processes that underlie PPCs following surgery. Second, in order to thoroughly evaluate the utility of these markers, we used a very strict validation framework that included discrimination ability, calibration, clinical decision curves, and model interpretability analysis. We discovered a strong synergistic effect between pleural metastasis and anastomotic leaking by additive interaction analysis; this finding has obvious implications for clinical risk classification. In order to facilitate the application of these findings in clinical practice and the translation of research findings into clinical implementation, we finally converted the risk factors into an easy-to-use nomogram tool.

However, there are certain drawbacks to this study as well. First, despite thorough internal validation, the results of this single-center retrospective investigation could be skewed by selection bias. Further validation of their generalizability is needed in large-scale, prospective, multicenter cohort investigations. Second, even though we included a wide variety of clinical and laboratory variables, the analysis might not have fully taken into account some potential confounding factors, such as more precise pulmonary function measures, patient performance status scores, or unrecorded concurrent medications. Additionally, after excluding postoperative length of stay to avoid reverse causality, the overall discriminative power (AUC) of the preoperative risk model was moderate. This reflects the inherent complexity of PPCs, which are influenced by multiple dynamic intraoperative and postoperative factors beyond baseline inflammation. Finally, in our retrospective database, we did not systematically record whether patients had a history of asthma/allergies or routine preoperative steroid use. These conditions may influence the levels of inflammatory mediators in patients. Nevertheless, the strong independent associations and calibration of the identified risk factors confirm their biological and clinical significance. Future studies might include dynamic monitoring of postoperative inflammatory marker patterns, which might more accurately identify the window of opportunity for the beginning of complications.

## 5 Conclusion

This work systematically evaluated the independent prognostic value of novel inflammatory markers and surgical factors for PPCs. Instead of merely creating a forecasting tool, this study highlighted the biological significance of preoperative Eosinophils and LMR. Ultimately, it provides clinicians with valuable insights to recognize biologically vulnerable patients early, facilitating customized, focused preventive tactics for those at the highest risk.

## Data Availability

The raw data supporting the conclusions of this article will be made available by the authors, without undue reservation.
